# The peptide symporter SLC15a4 is essential for the development of systemic lupus erythematosus in murine models

**DOI:** 10.1371/journal.pone.0244439

**Published:** 2021-01-14

**Authors:** Arna Katewa, Eric Suto, Jessica Hui, Jose Heredia, Jie Liang, Jason Hackney, Keith Anderson, Tuija M. Alcantar, Natasha Bacarro, Debra Dunlap, Jeffrey Eastham, Andres Paler-Martinez, Xin Y. Rairdan, Zora Modrusan, Wyne P. Lee, Cary D. Austin, Daniel Lafkas, Nico Ghilardi

**Affiliations:** 1 Dept. Biochemical and Cellular Pharmacology, Genentech, South San Francisco, CA, United States of America; 2 Dept. Translational Immunology, Genentech, South San Francisco, CA, United States of America; 3 Evercore ISI, New York, NY, United States of America; 4 Dept. Immunology, Genentech, South San Francisco, CA, United States of America; 5 Dept. Molecular Oncology, Genentech, South San Francisco, CA, United States of America; 6 Dept. Bioinformatics, Genentech, South San Francisco, CA, United States of America; 7 Dept. Molecular Biology, Genentech, South San Francisco, CA, United States of America; 8 Dept. Pathology, Genentech, South San Francisco, CA, United States of America; 9 gRED Animal Resources, South San Francisco, CA, United States of America; 10 Dept. Microchemistry, Proteomics, & Lipidomics, Genentech, South San Francisco, CA, United States of America; 11 DiCE Molecules, South San Francisco, CA, United States of America; University of Nebraska-Lincoln, UNITED STATES

## Abstract

Systemic Lupus Erythematosus (SLE) is a chronic autoimmune disease representing a serious unmet medical need. The disease is associated with the loss of self-tolerance and exaggerated B cell activation, resulting in autoantibody production and the formation of immune complexes that accumulate in the kidney, causing glomerulonephritis. TLR7, an important mediator of the innate immune response, drives the expression of type-1 interferon (IFN), which leads to expression of type-1 IFN induced genes and aggravates lupus pathology. Because the lysosomal peptide symporter slc15a4 is critically required for type-1 interferon production by pDC, and for certain B cell functions in response to TLR7 and TLR9 signals, we considered it as a potential target for pharmacological intervention in SLE. We deleted the *slc15a4* gene in C57BL/6, NZB, and NZW mice and found that pristane-challenged *slc15a4*^*-/-*^ mice in the C57BL/6 background and lupus prone *slc15a4*^*-/-*^ NZB/W F1 mice were both completely protected from lupus like disease. In the NZB/W F1 model, protection persisted even when disease development was accelerated with an adenovirus encoding IFNα, emphasizing a broad role of slc15a4 in disease initiation. Our results establish a non-redundant function of slc15a4 in regulating both innate and adaptive components of the immune response in SLE pathobiology and suggest that it may be an attractive drug target.

## Introduction

Systemic Lupus Erythematosus (SLE) is a chronic autoimmune disease characterized by multi-organ inflammation [1] with complex pathophysiology that involves both genetic risk factors and environmental triggers [2]. It is thought that inadequate clearance of apoptotic cells results in self-sustaining activation of the immune system, which involves the production of anti-nuclear antibodies, immune complex (IC) formation, and IC deposition to drive inflammation and damage in multiple organs, including the kidney. Nucleic acid-IC are known to activate the innate immune response through Toll-like receptors (TLR) 7 and 9 in plasmacytoid dendritic cells (pDC) and other cell types, driving production of type I interferon. Accordingly, a strong interferon signature metric (ISM), which correlates with SLE disease severity, is present in human patients [3, 4]. IFNα activity likely contributes to disease pathogenesis, because non-SLE individuals treated with recombinant IFNα for other reasons can develop an SLE like syndrome [5–7]. Furthermore, the use of a neutralizing antibody directed against the IFNαR (anifrolumab) resulted in a substantial therapeutic effect in a phase III clinical trial [8], even though it failed in an earlier trial with a different endpoint [9]. In addition to type I interferon, B cells have been firmly established as pathogenic drivers in SLE. Belimumab, a BAFF blocking antibody that impacts B cell survival, differentiation, and auto-antibody production [10], led to a significant reduction of the SELENA-SLEDAI score and represents the only new lupus medicine that has been approved in the last 50 years [11, 12]. Although another B cell targeting therapy, the CD20 targeting antibody rituximab, did not achieve statistically significant improvement in two clinical trials [13, 14], it is nevertheless used to treat the disease off-label [15], based in part on demonstration of potent therapeutic effects in a non-registrational trial [16]. Thus, based on existing human clinical data, a molecular target that impinges on the IFNα pathway and also affects B cells directly might be highly desirable therapeutic opportunity for this intractable disease.

Solute carrier family 15 (SLC15) are a group of five known proton coupled oligopeptide transporters [17, 18]. SLC15a1 and SLC15a2 are expressed on the plasma membrane and essential for uptake of oligopeptides in the intestine and the kidney, whereas SLC15a5 is predominantly expressed in the brain and appears to be associated with psychological resilience. SLC15a3 and SLC15a4 are found primarily in the endo-lysosomal membrane of immune cells, where they facilitate the translocation of histidine and bacterially-derived dipeptides such as muramyl dipeptide (MDP), a NOD2 ligand [19]. While they seem to be functionally indistinguishable, their cellular expression pattern differs. Slc15a3 is predominantly expressed in macrophages, whereas slc15a4 is most abundant in B cells and, especially, pDC. An unexpected function of slc15a4 was revealed in the context of an in vivo mutagenesis screen, which identified this gene as critically required for TLR9 induced IFNα production [20]. Subsequently, these authors demonstrated that slc15a4 deficient mice have normal counts of pDC; however, these pDC fail to secrete IFNα and have significantly reduced secretion of other inflammatory cytokines in response to TLR7 or TLR9 stimulation [20]. While it has recently been demonstrated that SLC15a4 recruits the adapter molecule TASL to provide a binding site for IRF5 [21], it has also been appreciated that the physiological role of SLC15a4 is dependent on transport function [22] and should thus be amenable to pharmacological targeting. Furthermore, it has been demonstrated that slc15a4 deficiency affects the luminal pH of lysosomes, mTOR signaling, and secretory granule homeostasis [22, 23].

Slc15a4 deficiency has physiological consequences, as slc15a4^-/-^ mice were unable to mount effective CD8 responses to lymphocytic choriomeningitis virus (LCMV) clone 13 and failed to clear the virus [24]. They were also resistant to dextran sulfate sodium (DSS) induced colitis, presumably due to an inability to transport nucleotide binding oligomerization domain containing 1 (NOD1) ligands into the cytosols of innate immune cells [25]. Furthermore, slc15a4 deficiency also led to resistance to disease in the Fas^lpr^ model of SLE [26], which is known to be heavily dependent on TLR7 signaling. Interestingly, slc15a4 deficiency also impaired B cells, in particular their production of IgG2c, and resulted in attenuated autoantibody production in mice injected with pristane [22]. These findings from rodent models are complemented by several GWAS studies that genetically implicate SLC15A4 in human SLE [27–32].

Based on this evidence, we hypothesized that slc15a4 might be a suitable drug target for SLE. Since pharmacologic inhibitors of sufficient quality are currently not available, we decided to employ genetically altered mice for validation of slc15a4 as a target for the treatment of SLE.

## Results

**Fig 1:**
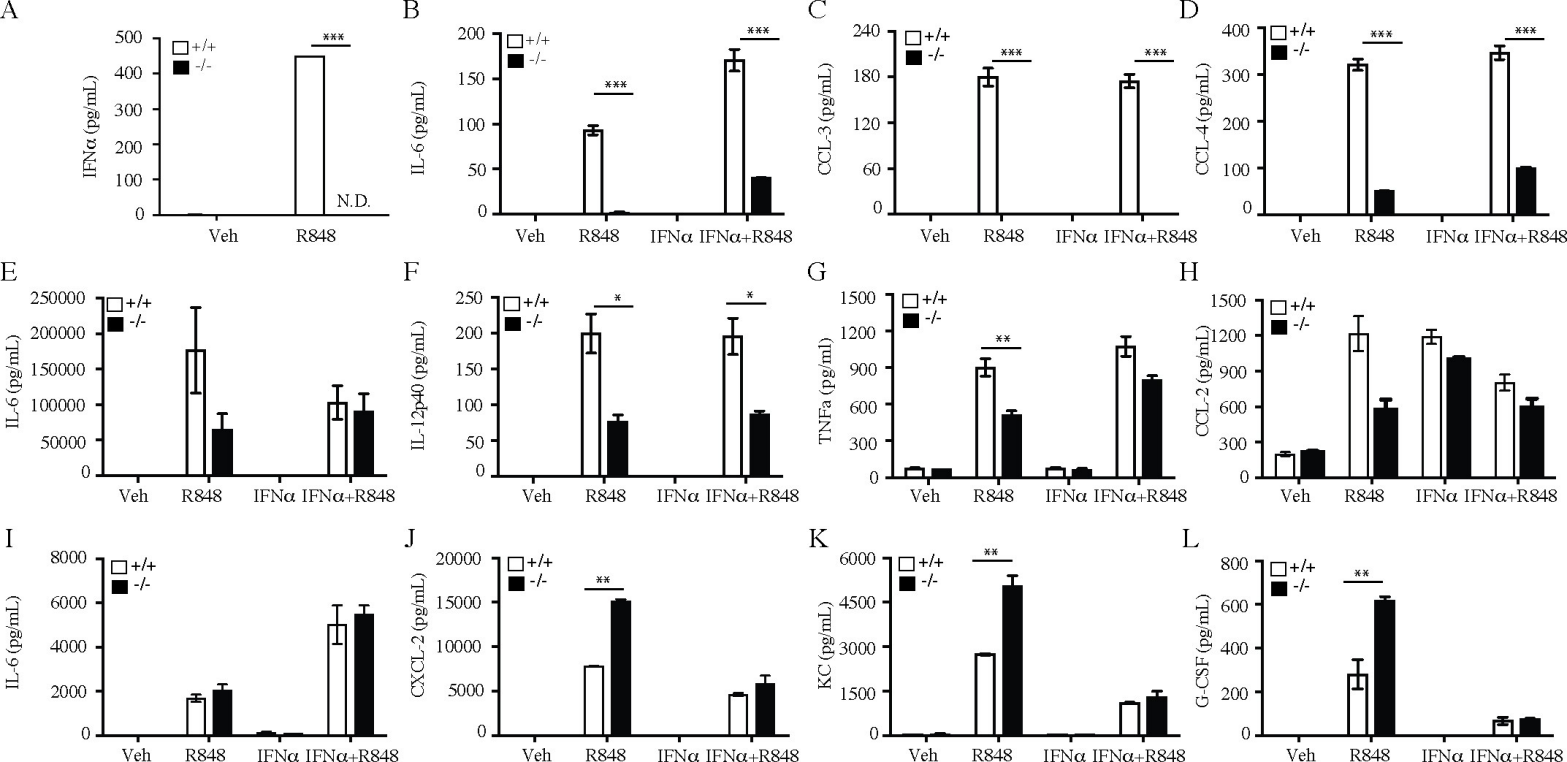
SLC15A4 regulates TLR7 induced inflammatory cytokine secretion in dendritic cells and macrophages. (A) Secretion of IFNα by bone marrow derived pDC in response to R848 stimulation (B-L) Cytokine/chemokine secretion by bone marrow derived pDC (B-D), myeloid DC (E-H), and macrophages (I-L) in response to stimulation with 1μg/ml of R848, or 5U/ml IFNα, or stimulation with both agents. Cytokine / chemokine levels in supernatants were assessed by Luminex (Bio-Rad platform) after 24 hours of stimulation and data is expressed as mean± SD for triplicate samples (n = 3). White bars, wild-type mice (C57BL/6); black bars, *slc15a4*^*-/-*^ mice (on C57BL/6 background). Comparisons were made between wildtype group and knockouts or heterozygous groups *p < 0.05, **p<0.005, ***p<0.0005.

As expected based on prior work [20], *slc15a4*^*-/-*^ pDC did not produce IFNα in response to stimulation with the TLR7 agonist R848 (Fig 1A). Production of other pDC derived cytokines and chemokines was also substantially, but not always completely reduced (Fig 1B and 1C). Consistent with relatively higher expression of slc15a3 in mDC and macrophages [33, 34], TLR7 induced cytokine / chemokine production was only partially compromised in myeloid DC from *slc15a4*^*-/-*^ mice (Fig 1E–1H), and seemingly unaffected or even enhanced in *slc15a4*^*-/-*^ macrophages (Fig 1I–1L). Thus, slc15a4 deficiency does not result in complete non-responsiveness to TLR7 stimulation, even though IFNα production by pDC is completely abolished. To determine whether reduction of TLR7 inducible factors was due to abolition of secondary, autocrine IFNα signaling, we added recombinant IFNα to the cultures. In general, addition of recombinant IFNα does not rescue the *slc15a4*^*-/-*^ defects in any cell type (Fig 1). Thus, slc15a4 deficiency modulates TLR7 signaling to varying degrees in different cell types.

**Fig 2:**
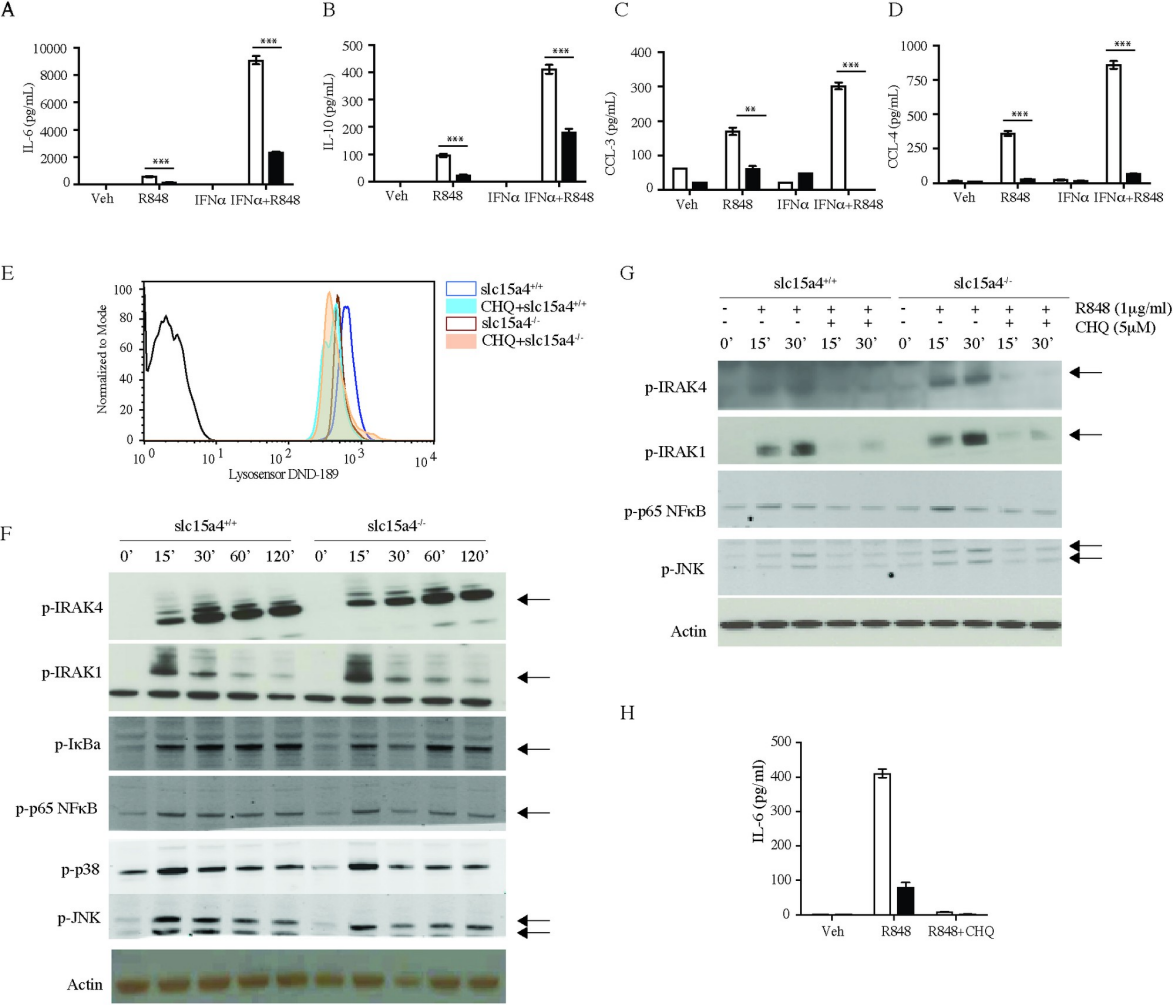
SLC15A4 regulates cytokine secretion but not NFκB phosphorylation in B cells in response to TLR7 stimulation. (A-D) Cytokine and chemokine levels in the supernatants of B cells stimulated with 1μg/ml of R848, or 5U/ml IFNα, or both agents for 72 hours were measured by Luminex (Bio-Rad platform). (E) lysosomal pH determined by lysosensor dye and expressed as histogram overlay of wild type B cells in absence (blue) or in presence of chloroquine (filled light blue) and slc15a4^-/-^ B cells in absence (brown) or in presence of chloroquine (filled light orange). (F-G) Splenic B cells were stimulated with 1μg/ml of R848 for indicated times with or without chloroquine, and phosphorylation of signaling molecules was assessed by western blot. (H) IL-6 levels in the supernatants of B cells stimulated with 1μg/ml of R848 with or without chloroquine. Data is expressed as mean± SD for triplicate samples (n = 3). White bars, wild-type mice (C57BL/6); black bars, *slc15a4*^*-/-*^ mice (on C57BL/6 background). *p < 0.05, **p<0.005, ***p<0.0005.

We next turned our attention to B cells, which have also been found compromised in *slc15a4*^*-/-*^ mice [22]. Similar to myeloid cells, cytokine and chemokine production elicited by TLR7 stimulation was reduced in B cells. Furthermore, while recombinant IFNα synergized with R848, it failed to correct the defect in *slc15a4*^*-/-*^ B cells (Fig 2A–2D). Since it had been hypothesized slc15a4 deficiency results in altered lysosomal pH due to accumulation of histidine, we assessed the lysosomal pH in B cells from wild type and slc15a4^-/-^ B cells using lysosensor dyes (Fig 2E). Although we observed a small increase in lysosomal pH in slc15a4^-/-^ B cells, it was not as substantial as the shift induced by chloroquine, and chloroquine could further increase the pH in slc15a4^-/-^ lysosomes. Accordingly, slc15a4 deficiency did not affect proximal TLR signaling, as illustrated by normal activation of IRAK4, IRAK1, IκBα, NFκB p65, p38 and JNK in *slc15a4*^*-/-*^ B cells (Fig 2F), whereas addition of chloroquine suppressed activation of these signaling molecules (Fig 2G) and completely abrogated R848 induced IL-6 production (Fig 2H).

Together, these results suggest that deficiency of slc15a4 does not alter NFκB and MAPK signaling downstream of TLR7 stimulation. This is consistent with the recent finding that slc15a4 binds to adaptor protein TASL which is essential of recruitment of IRF5 and cytokine production upon TLR7 stimulation without having any effect on NFκB and MAPK signaling [21]. Since slc15a4 deficiency affected TLR7 signaling in a restricted fashion, we next analyzed R848 stimulated B cells by RNASeq. We observed a decrease in overall gene expression in slc15a4^-/-^ B cells with a significant effect on interferon signature genes, but not NFκB dependent genes (S1A–S1L Fig). There was a significant decrease in expression of inflammatory cytokines and genes involved in plasma cell differentiation in the absence of slc15a4 (S1M, S1N Fig). B cells and myeloid cells were fully capable of responding to TLR4 (S1O, S1P and S2 Figs). We thus conclude that slc15a4 deficiency affects multiple immune cell types and leads to suppression of immune mediators elicited specifically upon TLR7 stimulation, including, but not limited to IFNα.

**Fig 3:**
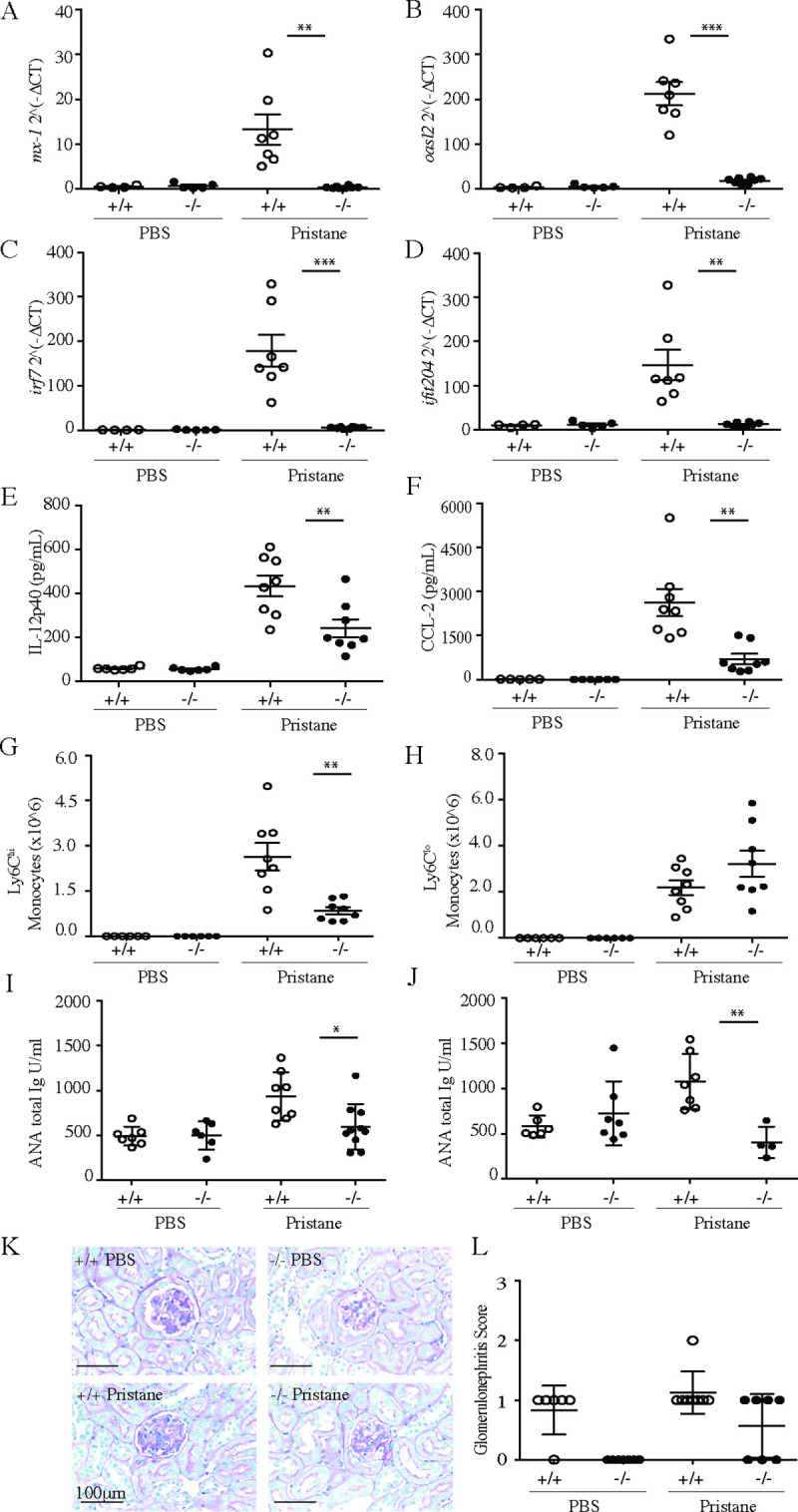
SLC15A4 deficient mice are protected in pristane induced lupus model. Two cohorts of 11-week-old wild-type mice (C57BL/6) and *slc15a4*^*-/-*^ mice (on C57BL/6 background) were injected intraperitoneally with either 500μl PBS or pristane. Two weeks post pristane administration, peritoneal lavage was collected from cohort 1, and expression of Interferon signature genes (A-D), secretion of IL12p40 (E) and CCL-2 (F), and number of inflammatory Ly6Chi monocytes (G-H) were assessed by qRT-PCR, Luminex and flow cytometry, respectively. In cohort 2, serum concentrations of anti-nuclear antibodies were assessed 4 months (I) and 9 months (J) after pristane administration. Renal glomerulonephritis was assessed by histological assessment of H&E stained sections after 9 months of pristane administration (K) and scored as described in the methods section (L), and plotted with group means ±SD. *p < 0.05, **p<0.005, ***p<0.0005, N = 7 animals per group (some mice had to be euthanized during study due to fight injuries).

Since TLR7 and IFNα are intimately linked to lupus pathogenesis [35–38], we next wanted to expand on prior results in the 2,6,10,14-tetramethylpentadecane (TMPD, also known as pristane) elicited murine model of lupus [22, 39]. In this model, a subset of Ly6C^hi^ monocytes represent a dominant source of IFNα [39]. We first evaluated the expression of interferon induced genes in peritoneal exudate cells two weeks after pristane administration and found that wild type mice mounted a strong interferon response. In contrast, expression of these genes in slc15a4^-/-^ mice was almost completely abrogated (Fig 3A–3D). IL-12p40 and CCL-2 levels in peritoneal exudate were partially reduced (Fig 3E and 3F). These findings were consistent with a strongly reduced accumulation of Ly6C^hi^ monocytes, but not Ly6C^mid^ monocytes (Fig 3G and 3H). Furthermore, and consistent with prior results published by others [22], we also observed a reduction in anti-nuclear antibodies 4 and 9 months after pristane injection (Fig 3I and 3J). However, particularly in C57BL/6 mice, this model does not elicit strong renal pathology, and hence did not allow us to evaluate improvement of glomerulonephritis in the absence of SLC15A4 (Fig 3K and 3L).

**Fig 4:**
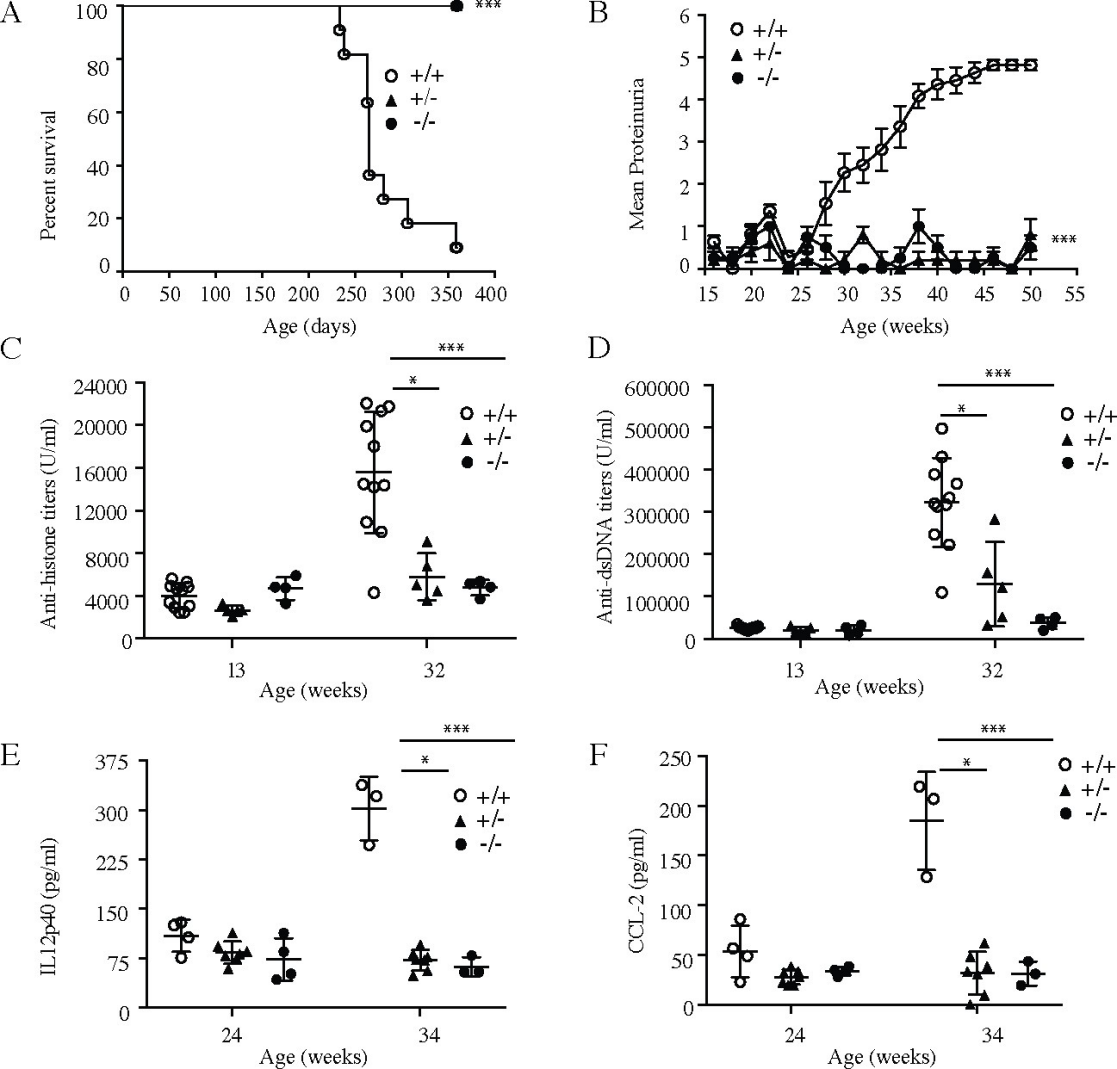
Lack of SLC15A4 protects mice from developing spontaneous lupus phenotype in NZB/W F1 mice. NZB/W F1 *slc15a4*^+/+^, *slc15a4*^+/-^, and *slc15a4*^*-/-*^ mice were enrolled at an age of 13 weeks and monitored every two weeks. Survival (A) and proteinuria (B) were monitored throughout the study. Serum was evaluated at indicated times for the presence of anti-dsDNA antibodies (C), anti-histone antibodies (D), IL12p40 (E), and CCL-2 (F). Comparisons were made between wildtype group and knockouts or heterozygous groups *p < 0.05, **p<0.005, ***p<0.0005, N = 4–10 mice per group.

**Fig 5:**
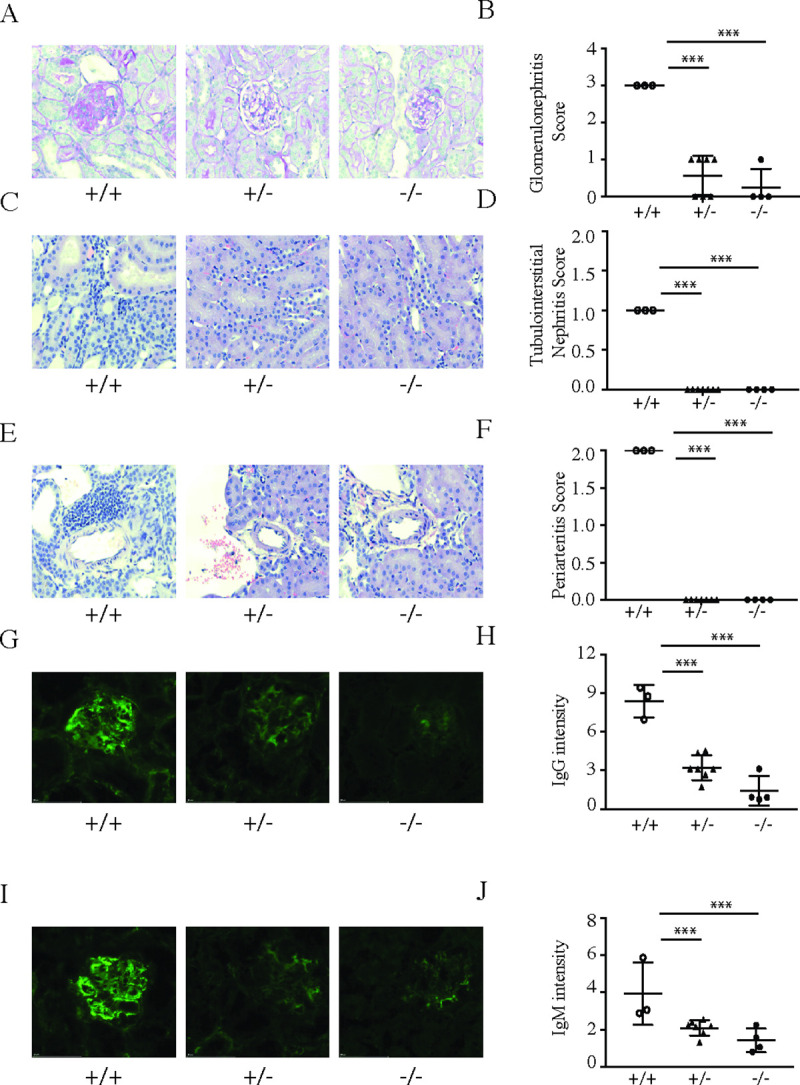
Lack of SLC15A4 protects mice from developing spontaneous lupus nephritis in NZB/W F1 mice. NZB/W F1 *slc15a4*^+/+^, *slc15a4*^+/-^, and *slc15a4*^*-/-*^ mice were euthanized at the age of 36 weeks. H&E and PAS staining was carried out on formalin fixed tissues to assess renal disease severity by comparing severity scores of glomerulonephritis (A, B), tubulointerstitial nephritis (C, D) and periarteritis (E, F). Immune-complex deposition in kidney was assessed in frozen tissue sections by staining for IgG (G,H) and IgM (I,J) and quantified using FITC/AF488 integrated intensity in renal cortex. Examples (A, C, E, G, I) and quantification over several samples (B, D, F, H, J) is shown. Data is plotted with group means ± SD; individual data points represent results from two kidney sections on one slide from an individual mouse. *p < 0.05, **p<0.005, ***p<0.0005.

In order to test the consequences of slc15a4 deletion in a model more reflective of human SLE with severe nephritis, we employed CRISPR technology to introduce deletions into the NZB and NZW strains of lupus prone mice (S3A and S3B Fig). Although distinct from each other, both alleles resulted in complete abrogation of slc15a4 protein production (S3C Fig). Slc15a4^+/+^, slc15a4^+/-^, and slc15a4^-/-^ littermates were produced by intercrossing the two alleles in their respective genetic backgrounds. Functional assays using B cells and pDC from wild type, heterozygous and knockout mice recapitulated the defects observed in the C57BL/6 background, with heterozygous mice showing an intermediate response (S3D–S3G Fig). At 13 weeks of age (before disease onset), serological analysis did not reveal any differences in the levels of immunoglobulin isotypes in the serum (S4 Fig). Consistent with historical data, wild-type animals spontaneously developed disease, as exemplified by mortality, proteinuria, autoantibody production, and elevated serum concentrations of IL-12p40 and CCL-2 (Fig 4). Furthermore, serum concentrations of several Ig isotypes increased by 32 weeks of age (S4 Fig). These results demonstrate that the genetic engineering and rederivation processes did not abrogate the ability of NZB/W F1 mice to develop spontaneous lupus disease. In stark contrast, *slc15a4*^*-/-*^ NZB/W F1 animals were completely protected from all parameters of disease (Fig 4). To our surprise, heterozygous animals also failed to develop disease within the time frame of the experiment, suggesting that a 50% reduction in slc15a4 expression is sufficient for protection from spontaneous lupus development. A non-significant increase in autoantibody production was noted in heterozygous animals (Fig 4C and 4D). Histological analysis (Fig 5A–5F) and quantification of immune complex deposition in the kidney (Fig 5G–5J) further confirmed the absence of disease in heterozygous and knockout animals.

**Fig 6:**
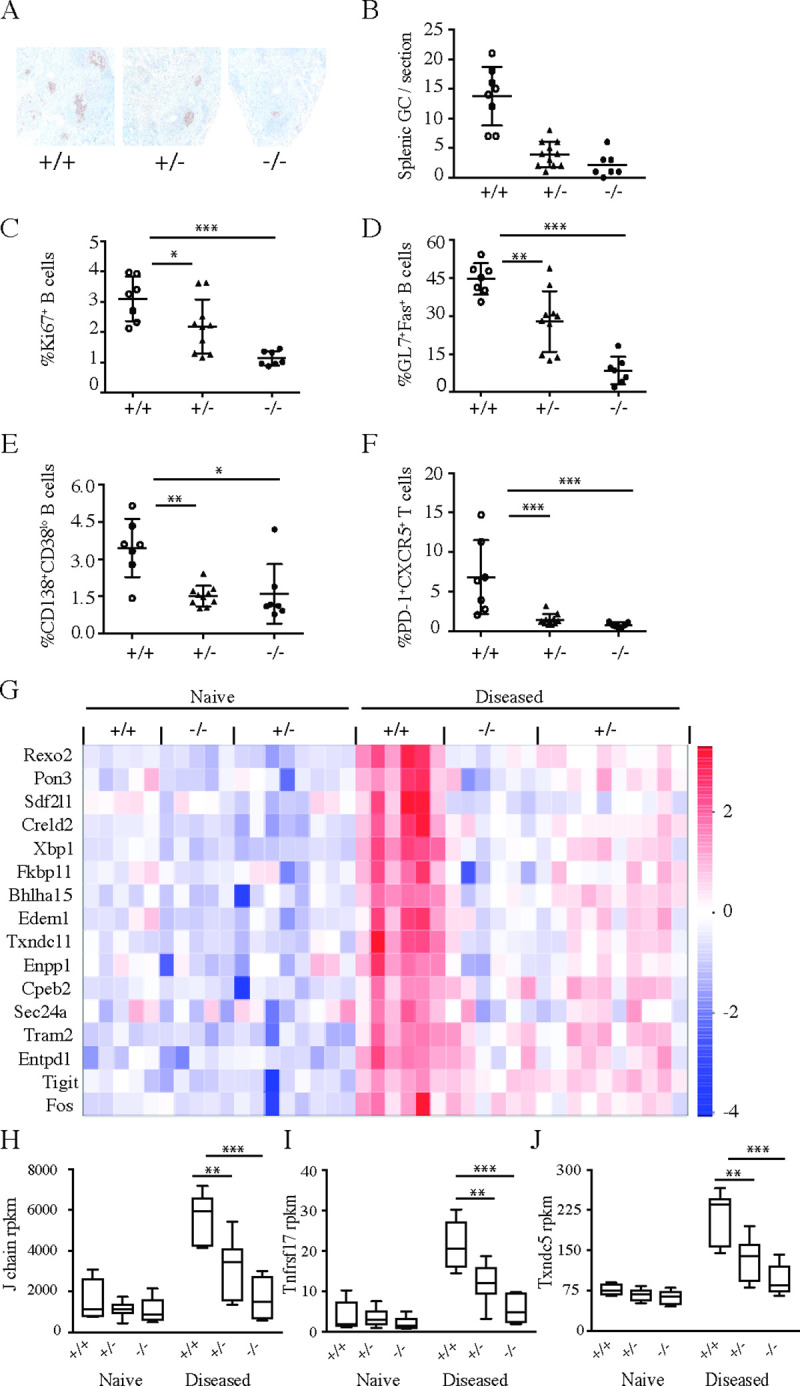
Lack of SLC15A4 causes a defect in development of germinal centers and differentiation of antibody secreting cells in NZB/W F1 mice. NZB/W F1 *slc15a4*^+/+^, *slc15a4*^+/-^, and *slc15a4*^*-/-*^ mice were euthanized at the age of 36 weeks. Development of splenic germinal centers was assessed by PNA-lectin staining and immunohistochemistry in formalin fixed spleens (A) and quantified for all mice in the experiment (B). The phenotype of splenic lymphocyte population from individual mice was determined by flow cytometry and expressed as mean ± SD. Symbols represent individual mice and depict frequencies of proliferating B cells (B220^+^Ki67^+^) (C), germinal center B cells (GL7^+^Fas^+^) (D), splenic plasma cells (CD138^+^CD38^lo^) (E) and follicular T helper cells (PD1^+^CXCR5^+^) (F). Gene expression changes in spleens were assessed by RNA sequencing. Specifically, selected transcripts associated with plasma cell generation and induced upon disease development (log2-fold change > 2, p < 0.05) were significantly reduced in heterozygous and *slc15a4*^*-/-*^ mice (G). Representative box plots of genes expressed by antibody secreting cells including J chain (H), tnfrsf17 (I) and txndc5 (J) are shown as mean ± SD. Comparisons were made between wildtype group and knockouts or heterozygous groups *p < 0.05, **p<0.005, ***p<0.0005, N = 7 per group.

The above results suggested that slc15a4 deficiency resulted in an early interruption of disease pathogenesis. We therefore next analyzed the spleens of 36 week-old mice. Knockout NZB/W F1 animals displayed dramatically reduced numbers of germinal centers (Fig 6A and 6B), proliferating (Ki67^+^) B cells (Fig 6C), germinal center B cells (Fig 6D) and plasma cells (Fig 6E). Consistent with the reduction of germinal centers, the percentage of T follicular helper (T_FH_) cells was also drastically diminished (Fig 6F). Gene expression analysis in spleens by RNA-seq revealed that *slc15a4*^*-/-*^ NZB/W F1 mice failed to upregulate genes associated with plasma cell generation (Fig 6G and 6H). We also noted that heterozygous mice displayed an intermediate phenotype in the spleen, which was consistent with a trend towards autoantibody development (Fig 4C and 4D). This intermediate phenotype suggests that disease development may not be completely abrogated, but strongly delayed. Long term studies will be required in order to determine if *slc15a4*^*+/-*^ NZB/W F1 mice are still capable of developing lupus like disease.

**Fig 7:**
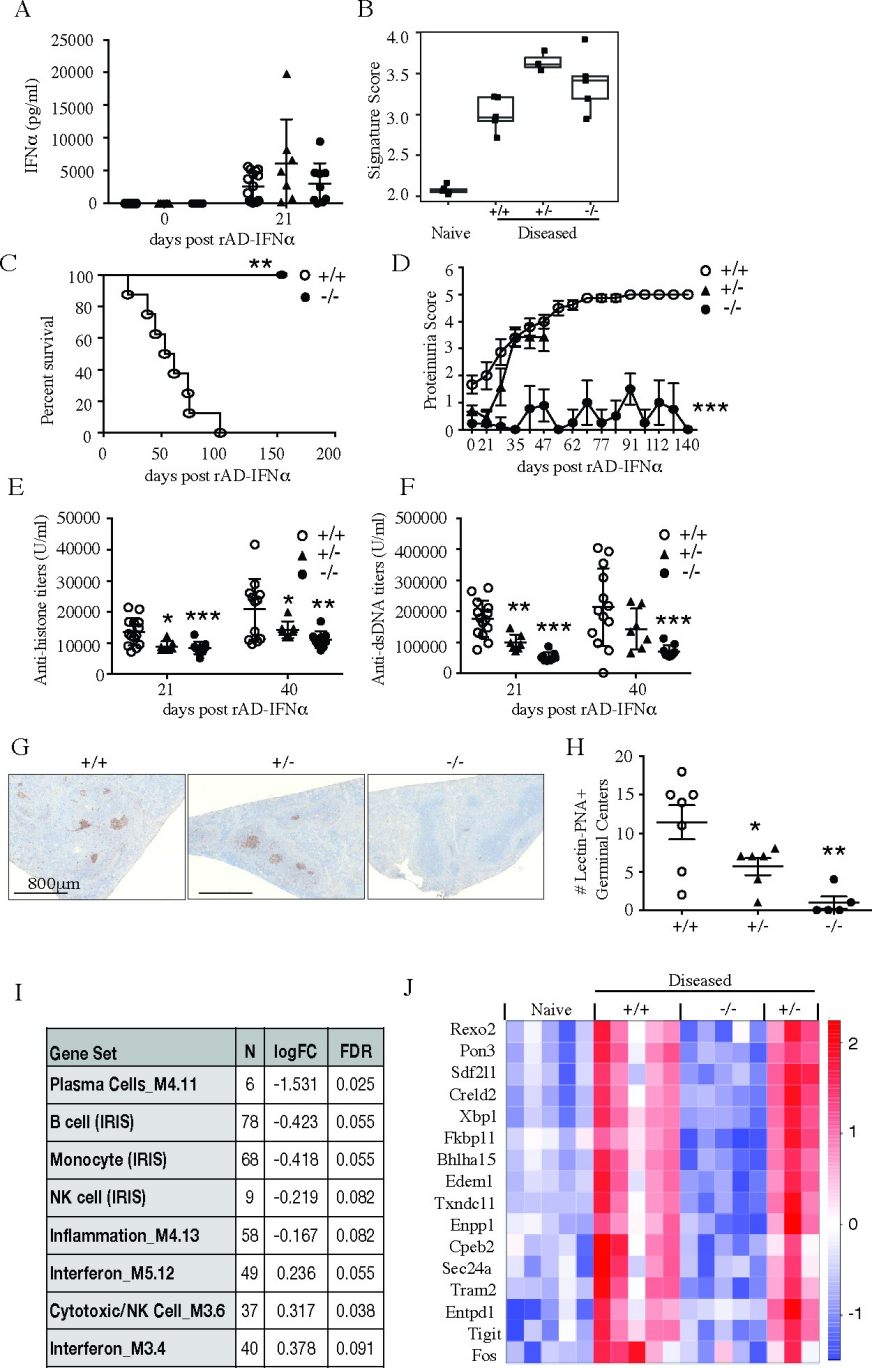
SLC15A4 deficient mice are protected from IFNα accelerated lupus disease development in NZB/W F1 mice. 14–15 week-old NZB/W F1 *slc15a4*^+/+^, *slc15a4*^+/-^and *slc15a4*^*-/-*^ mice were injected intravenously with 1.2x10^8^ pfu IFNα rAd5- IFNα. Three weeks post rAd-IFNα administration, serum IFNα levels were assessed by ELISA (A) and interferon gene signature score was assessed in spleen after 7 weeks (B). Mice were regularly monitored for survival (C) and proteinuria (D) rAd-IFNα administration. Serum anti-histone antibody (E) and anti-dsDNA antibody (F) titers were assessed by ELISA 3 weeks and 6 weeks post rAd-IFNα administration. Data is expressed as mean ± SD and each symbol represents an individual mouse in respective groups. Germinal center formation was assessed by PNA staining in spleen after 7 weeks; representative immunohistochemistry sections (G) and numbers of PNA^+^ germinal centers expressed as mean ± SD (H) are shown. Gene set enrichment analysis was performed using pre-defined immune gene modules after 7 weeks. Gene sets that showed significant enrichment (p<0.01) are shown with log fold change and FDR (I). Transcripts associated with antibody-secreting cell generation were significantly reduced in the absence of slc15a4 and visualized here as heatmap of select antibody-secreting cell-specific transcripts that were induced with disease (log2-fold change > 2, p < 0.05) (J). Comparisons were made between wildtype group and knockouts or heterozygous groups *p < 0.05, **p<0.005, ***p<0.0005, N = 5 per group.

In this model, induction of ISM was so limited that we could not reliably detect a decrease of ISM gene expression in *slc15a4*^*-/-*^ NZB/W F1 mice. However, since IFNα signature is known to precede disease onset in NZB/W F1 model [40, 41], it was still formally possible that low induction of an interferon response was both required for disease development and dependent on slc15a4. We therefore hypothesized that disease might be rescued if *slc15a4*^*-/-*^ NZB/W F1 mice were treated with an adenovirus encoding IFNα (rAd-IFNα). In order to maximize the amount of data we could collect from a limited cohort of mice (see methods), we designed a combined survival and biomarker study (S5 Fig). All mice were injected intravenously with 1.2x10^8^ pfu of rAd-IFNα, which resulted in comparable levels of IFNα serum levels and expression of interferon signature genes (Fig 7A and 7B). In spite of IFNα restoration, *slc15a4*^*-/-*^ mice did not develop any overt signs of disease (Fig 7C and 7D), nor did they produce a germinal center response or autoantibodies (Fig 7E–7H). RNA sequencing and gene set enrichment analysis showed a similar decrease in plasma cell reduction as observed in the spontaneous model (Fig 7I and 7J). However, unlike in the spontaneous model, rAd-IFNα injected heterozygous slc15a4^*+/-*^ NZB/W F1 mice developed proteinuria, germinal centers, autoantibodies and a plasma cell gene signature similar to slc15a4^*+/+*^ NZB/W F1 mice, and were thus no longer fully protected (Fig 7D–7J).

**Fig 8:**
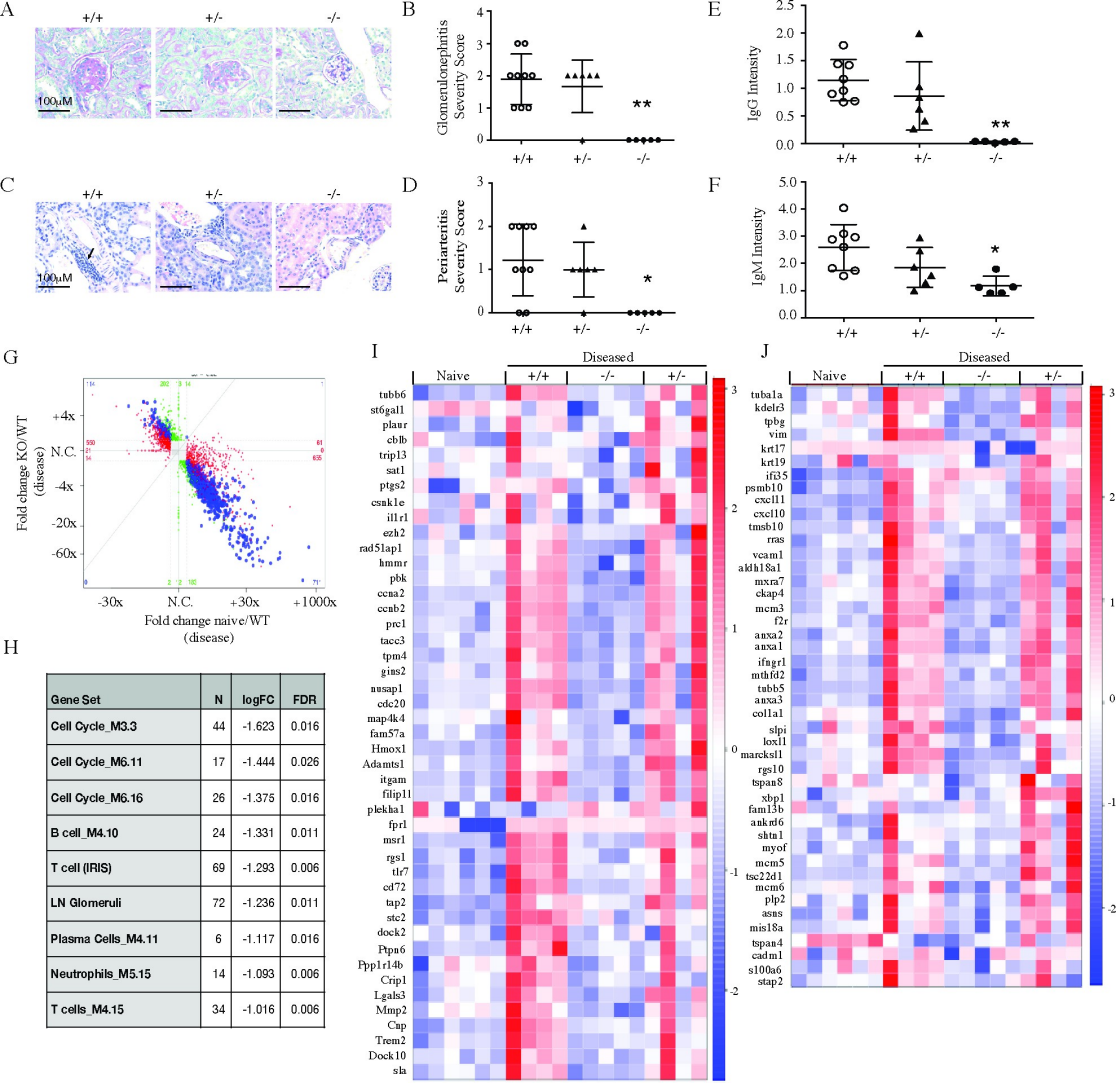
SLC15a4 deficient NZB/W F1 mice are protected from IFNα accelerated lupus nephritis. 7 weeks post rAd-IFNα administration, NZB/W F1 *slc15a4*^+/+^, *slc15a4*^+/-^and *slc15a4*^*-/-*^ mice were euthanized and kidneys were collected to assess renal disease severity by histology and RNA sequencing. (A-D) Representative images and quantification of histological scoring for glomerulonephritis (A, B) and periarteritis (C, D) are shown. (E-F) Quantification of IgG (E) and IgM (F) immune complex deposition. Data is expressed as mean± SD. (G) RNA sequencing analysis was performed on kidney RNA. Fold changes between KO and WT at the disease timepoint are compared to those between naïve WT and disease WT animals. Each data point represents a gene. Red, green and blue dots represent genes that are significant only in naive versus diseased condition in wildtype (red), only in knockout versus diseased wildtype (green) or both (blue). (H) Gene set enrichment analysis of pre-defined immune and human LN ortholog gene modules are shown. (I-J) Effect of slc15a4 on human LN ortholog genes previously identified to be up-regulated in the glomerulus (I) or tubulointerstitium (J) of nephritic kidneys from human LN patients (see Material and Methods for details). Genes that showed individual significant changes in absence of slc15a4 are indicated (≥1.5 fold difference between groups with an adjusted p-value <0.01). Each column in the heatmap represents one animal within each group. *p < 0.05, **p<0.005, ***p<0.0005. N = 5–7 per group.

Analysis of the kidneys of rAd-IFNα-challenged, *slc15a4*^*-/-*^ NZB/W F1 mice confirmed absence of disease as evaluated by histology (Fig 8A–8D) and immune complex deposition (Fig 8E and 8F), whereas slc15a4^*+/-*^ NZB/W F1 mice were only minimally protected. Transcriptional profiling of kidney RNA showed a tight correlation between genes that are induced in disease and genes that are slc15a4 dependent (Fig 8G). To draw comparison to human lupus nephritis, we evaluated expression of murine orthologs of human gene sets that are enriched in human LN samples [42] (Fig 8H), and found nearly complete absence of genes normally found in diseased glomeruli (Fig 8I) or tubulointerstitium (Fig 8J). In summary, *slc15a4*^*-/-*^ NZB/W F1 mice were completely protected from lupus even in a situation where IFNα was delivered exogenously, whereas slc15a4^*+/-*^ NZB/W F1 mice developed the disease in this setting.

## Discussion

SLC15a4 is known to be essential for TLR7 and TLR9 dependent secretion of IFNα by pDCs and for inflammatory cytokine production by B cells. Here we show that slc15a4 not only regulates TLR7/9 responses in these two cell types, but is also partially required for secretion of proinflammatory cytokines by myeloid dendritic cells. This result suggests that slc15a4 may not be functionally redundant with slc15a3, which is readily expressed in mDC. Furthermore, the reduction of proinflammatory cytokines is not due to a secondary effect of loss of IFNα in absence of SLC15a4, since addition of exogenous IFNα did not rescue the phenotype of slc15a4 deficient B cells, PDCs and myeloid DCs. It was shown previously that Slc15a4 deficient B cells have an altered endo-lysosomal luminal pH that might directly impact luminal TLR signaling [23], and we also observed a slight change in the lysosomal pH of slc15a4^-/-^ B cells. However, we did not see any defect in the phosphorylation of proximal cytosolic signaling molecules or NFκB p65 in B cells upon TLR7 stimulation. Our observations are consistent with those made recently by Heinz et al, who demonstrated that the SLC15a4/TASL complex is critical for IRF5 dependent, but not NFκB or MAPK signaling in response to TLR7 signaling in immune cells [21].

We further evaluated slc15a4 as a molecular target for SLE in two murine models. In the absence of a high-quality tool compound to inhibit its function, we focused our efforts on the analysis of genetically altered mice. First, we studied the consequences of slc15a4 deletion in pristane induced lupus in the C57/BL6 background. This model recapitulates the induction of IFNα dependent genes, which is observed in about two thirds of all SLE patients [3, 4]; however, it often fails to develop high titers of autoantibodies and substantial kidney disease, particularly in the C57/BL6 background [43]. Slc15a4 deficiency nearly quantitatively suppressed the induction of ISM, and resulted in diminished expression of IL-12p40 and CCL-2 and reduced abundance of Ly6C^hi^ monocytes. However, autoantibody production was poorly induced, and thus we could only evaluate ANA, but not anti-nRNP antibodies. Furthermore, given the minimal induction of renal disease, we were unable to establish a potential therapeutic benefit conferred by slc15a4 deficiency in this model. Thus, we conclude from the pristane model that slc15a4 deficiency has a similar effect on ISM reduction as blockade of the interferon pathway, which has been investigated previously [44] and is a clinically validated target [8, 9].

To provide a better experimental system, we decided to delete slc15a4 from the germline of both NZB and NZW strains using CRISPR mediated gene targeting. To our knowledge, our study represents the first attempt to genetically modify this model. Prior to our experiments, individual loci have been bred into other backgrounds, for example NZM 2328 [45], which could then be intercrossed with gene deficient mice on the C57BL/6 background. In one example, NZB 2328 mice were crossed with IFNαR deficient animals to demonstrate that disease is IFNα dependent [46]. However, only direct modification of genes in NZB and NZW strains allows for assessment of pathways in the NZB/W F1 model of lupus. Using CRISPR technology, we generated two alleles, both of which resulted in complete absence of slc15a4 protein production. Importantly, the wild-type siblings of the targeted mice still developed disease, indicating that the disease phenotype can be maintained even though the mice are re-derived during the engineering process and my thus harbor altered microflora.

Our experiments with NZB/W F1 mice validated the importance of slc15a4 for lupus pathogenesis and yielded some surprising results. First, spontaneous disease development was effectively abrogated in *slc15a4*^*-/-*^ mice. We could not find any markers of disease activity, strongly suggesting a prominent role for slc15a4 in disease initiation. Furthermore, although it was previously well established that *slc15a4*^*-/-*^ mice are unable to mount a type I interferon response to TLR7 stimulation, disease was still completely absent when *slc15a4*^*-/-*^ NZB/W F1 mice were injected with rAd-IFNα. This result suggests that slc15a4 performs disease-relevant functions other than to allow for IFNα production, which is consistent with its demonstrated roles in B cell biology, particularly the production of disease relevant IgG2a/c isotype antibodies. However, since the expression of adenoviral IFNα is of limited duration, it remains a formal possibility that the role of slc15a4 in lupus is indeed solely to allow for IFNα production. We consider this unlikely, because we observed substantial deficiency in autoantibody production on day 21, a time point at which we still measured substantial amounts of IFNα in the serum.

In the spontaneous model, heterozygous mice were protected from proteinuria and death to the same extent as knockout animals, at least for the first year of their life. Sub-clinically, heterozygous mice began to show signs of slightly elevated autoantibodies and immune complex deposition in the kidney towards the end of the study, suggesting that they might ultimately develop disease if the experiment were run for the entire lifetime of these mice. In support of this interpretation, heterozygous mice did develop renal disease when injected with rAd-IFNα. Thus, heterozygous mice are not fully protected, but the loss of one allele of slc15a4 appears to attenuate spontaneous disease development substantially.

Our study has two important limitations. First, while our results establish that slc15a4 is required for disease initiation, we cannot determine the consequences of therapeutic intervention using our genetically modified animals. It has been demonstrated that the function of slc15a4 is transport dependent, because a transport-dead mutant failed to rescue the knockout phenotype [22]. Furthermore, the serendipitous discovery that certain drugs are strong inhibitors of slc15 family members [47] suggests that it should be possible to develop pharmacological inhibitors for slc15a4. However, to date, no such inhibitors are available, precluding experiments aimed at testing the consequences of therapeutic intervention. The recent finding that SLC15A4 interacts with the adaptor protein TASL to recruit and activate IRF5 [21] provide essential insight towards developing inhibitors that can potentially block this interaction to modulate TLR7 signaling and SLE pathogenesis.

In summary, we demonstrate that the development of lupus is dependent on the presence of slc15a4 in two different murine models. Our results support a role of slc15a4 that goes well beyond allowing for IFNα production. We show that slc15a4 critically regulates both innate and adaptive immune responses essential for development of SLE. Thus, we regard slc15a4 as a promising target for therapeutic intervention in this indication.

## Methods and materials

### Mice

*slc15a4*^*-/-*^ mice on the C57/BL6 background were described previously [19]. Slc15a4 knockout alleles in NZB and NZW strains were generated by CRISPR mediated gene targeting [48, 49]. For NZW mice, a single guide RNA (sgRNA1, GCGGCCTTCTACGGCGTCA) target and protospacer adjacent motif (PAM) was identified within exon 1 of Slc15a4 (ENSMUST00000031367.14). For NZB mice, a second sgRNA in exon 3 was identified (sgRNA2, CGTGGAGGGCCGTTCACAG) and used in combination with sgRNA1. We used a CRISPR design tool (Benchling) that uses the algorithm described by Hsu et al. [50] to provide ‘MIT’ specificity scores and off-target lists for each sgRNA. We chose to assess the top 11 predicted off-target loci for each sgRNA. The protocol for embryo microinjection and downstream analysis of G0 and G1 founder animals has been described previously [51]. In brief, 25 ng/μl Cas9 mRNA (Thermo Fisher; A29378) and 13 ng/μl each sgRNA (Synthego) were microinjected in the cytoplasm of NZW or NZB fertilized embryos. For NZW, a single sgRNA used relied on indel formation and a resulting frame-shift, which was identified using amplicon sequencing. A founder NZW mouse with an 8bp deletion (GACGCCGT) and lack of off-target activity was used to generate the line (8219). For NZB, a two sgRNA approach was used to delete a genomic region including part of exon 1 through exon 3 to truncate Slc15a4 and result in a frame-shifted protein. The founder NZB mouse used to generate the line (8021) harbored a 12609bp deletion allele (chr5: 127604626–127617234) and lacked off-target activity, identified initially via PCR (exon 3 forward + exon 1 reverse primers) and subsequently confirmed by NGS amplicon sequencing. Founders were selected for mating with NZW or NZB mice for germline transmission of the gene edited chromosome. Subsequent analysis of genomic DNA from G1 pups was used to confirm germline transmission of the targeted gene and the absence of off-target hits elsewhere in the genome. Male and female mice from colonies 8219 (NZW) and 8021 (NZB) were intercrossed to generate NZB/W F1 mice for spontaneous and IFNα accelerated lupus models. This genetic approach technically resulted in two kinds of heterozygous animals, and the knockouts are in fact compound heterozygotes. Since we could not discern any differences between the two kinds of heterozygous mice, we opted to pool the heterozygous mice for analysis, and we use the standard +/+, +/-, and -/- terminology to designate wild-type, heterozygous, and knockout animals, respectively. Breeding yielded offspring at the expected mendelian ratios, but was generally inefficient; we thus designed our experiments in such a way to derive maximal information from the limited number of mice we had available.

All animals were bred and housed at Genentech under specific pathogen free conditions. Only female mice were used in these experiments. All animal procedures were conducted under a protocol approved by the Institutional Animal Care and Use Committee at Genentech, and were performed in accordance with the *Guide for the Care and Use of Laboratory Animals*. All research personnel performing animal studies were trained by Genentech’s Animal Resource staff.

### Primary cell isolation and in vitro stimulation

Plasmacytoid dendritic cells, myeloid dendritic cells and macrophages were generated from bone marrow progenitor cells from age- and sex-matched wild type and *slc15a4*^*-/-*^ mice. Bone marrow cells were cultured for 10 days in the presence of 100ng/ml recombinant rmFLT3L, with supplementation of fresh media on day 5. CD11c^hi^, CD11b^lo^B220^hi^ pDC were purified by FACS sorting, resulting in 98% purity. Bone marrow derived dendritic cells (BMDC) were generated by culturing bone marrow cells in the presence of 10ng/ml of GM-CSF for 7 days, with supplementation of fresh media on day 3. Bone marrow derived macrophages (BMDM) were generated by culturing bone marrow cells in the presence of 10ng/ml of M-CSF for 7 days, with supplementation of fresh media on day 3. B cells were isolated from spleens of wild type and *slc15a4*^*-/-*^ mice using Myltenyi Biotech’s untouched B cell isolation kit and following the manufacturer’s protocol, which resulted in isolation of >95% purity as assessed by CD19 staining. B cells were stimulated for 72hrs, whereas plasmacytoid dendritic cells, myeloid dendritic cells and macrophages were stimulated for 24 hours with either 1μg/mL R848 or 5U/ml IFNα, or both agents in the presence or absence of chloroquine. B Cells were stimulated on the day of splenic isolation, BMDM and BMDC were stimulated on day 8, and pDC were stimulated on day 10 after sorting. Cytokine secretion was measured in the cell culture supernatant using Luminex.

### Induction of lupus with pristane

Two separate cohorts of mice were set up. Cohort 1 consisted of 11 week-old female mice and was used for the assessment of acute response. Cohort 2 consisted of 11 week-old male mice and was used for evaluation of autoantibody development proteinuria. Mice were injected intraperitoneally with either 500 μL of 2,6,10,14-tetramethylpentadecane (TMPD/pristane) or 500 μL of saline. To assess the acute response, two weeks post TMPD administration, cohort 1 was euthanized and peritoneal exudate was collected by needle aspiration and centrifuged. Cell pellets were used for FACS for inflammatory monocytes and RNA analysis of interferon signature genes, whereas supernatants were used for the measurement of cytokines and chemokines by Luminex method. Mice from cohort 2 were monitored for proteinuria every month for 9 months, and blood was collected at indicated times for the presence of serum anti-nuclear antibodies by ELISA. At 9 months post TMPD injection, mice from cohort 2 were euthanized, and kidneys were histologically analyzed for the presence of glomerulonephritis.

### NZB/W F1 model of lupus

Female NZB/W F1 mice were aged until they spontaneously developed lupus-like phenotypes. Viability and proteinuria were monitored starting at 15 weeks of age. Multiple cohorts of F1 mice were maintained with n = 4–10 per genotype, based on availability of F1 mice from the NZB x NZW breeding colony. In some experiments, mice were continuously monitored for survival and proteinuria for over a duration of either 52 weeks in case of spontaneous model or 20 weeks for IFNα accelerated model. Other cohorts were euthanized as soon as 80% of wild type F1 mice reached proteinuria levels of 300mg/dl, and tissues were collected to evaluate kidney pathology, splenic germinal center formation, cellular phenotyping in the spleen and RNA seq analysis of spleen and kidney. Lupus nephritis (LN) was accelerated in 14 to 15-week-old NZB/W F1 mice (n = 4–8 per genotype) by injecting IV 1.2x10^8^ IFNα rAd5-CMV (rAd-IFNα) plaque forming units (pfu) (generated by the Center for Cell & Gene Therapy, Baylor College of Medicine, TX). This treatment typically results in elevated serum IFNα levels of 10 ng/mL 3 weeks after injection. At 3 weeks post rAd-IFNα injection, mice were monitored for viability, proteinuria and autoantibody development. Similar to the spontaneous model, we maintained 2 separate cohorts to monitor either survival and proteinuria development or tissue analysis.

### Animal care and monitoring for survival study

Given that NZB/W F1 female mice succumb to disease over time resulting in mortality, all experimental animals were monitored by Study Investigator as well as Genentech’s Animal Resource staff. Monitoring by study investigator was carried out twice weekly for checking body weight and clinical condition. Frequency was increased if needed as directed by Genentech’s Vet staff due to appearance of adverse effects including excessive weight loss, dehydration, hunched posture, lethargy and loss of body condition. NZB/W mice that showed more than 35% body weight loss were monitored three times per week. As disease progressed in NZB/W F1 mice, unless found dead, as per Genentech’s Animal Resource and IACUC guidelines and Vet Staff’s guidance, if any of the mice exhibited body condition score of 2 or less (emaciated, severely hunched posture, severe lethargy or underconditioned with advanced muscle wasting) out of 5 were euthanized immediately.

Daily basic animal welfare monitoring was carried out by Genentech’s Animal Care and Vet staff.

### Proteinuria and survival scoring

Proteinuria was determined every two weeks by colorimetric measurement using dipstick Multistix 10 SG on a Clinitek Status Analyzer (Siemens). Urine protein levels were scored as trace = 0, 30 mg/dl = 1, 100 mg/dl = 2, 300 mg/dl = 3, >300 mg/dl = 4 and death = 5.

### Histology and immunohistochemistry and scoring

To evaluate renal pathology and splenic GC, kidneys and spleens were formalin-fixed and paraffin-embedded using routine methods. Four-micron tissue sections were stained and microscopically evaluated. Periodic acid–Schiff (PAS) stained kidney sections were scored for glomerulonephritis severity on a 4 point semiquantitative scale: “0”–normal or mild global lesions in <50% of glomeruli, “1”—global lesions in >50% of glomeruli, <20% of which are severe (defined as >1 segment with <3 patent capillaries), “2”—global lesions in >50% of glomeruli, 20–80% of which are severe, and “3”—>80% of glomeruli with severe global lesions. Hematoxylin and eosin (H&E) stained kidney sections were scored for periarteritis on a 4 point semiquantitative scale: “0”–no significant inflammation, “1”–sparse or focal periarterial inflammatory infiltrates, “2”–occasional moderate sized non-circumferential perivascular inflammatory infiltrates, and “3”–frequent large perivascular inflammatory infiltrates, often circumferential, and for tubulointerstitial nephritis on a 4 point semiquantitative scale: “0”–no significant inflammation, “1”–mild or focal inflammation not expanding the tubulointerstitium, “2”–occasional moderately sized inflammatory cell collections expanding the tubulointerstitium, and “3”–frequent large sized inflammatory cell collections expanding the tubulointerstitium. Splenic GC were detected by immunohistochemistry after Target antigen retrieval (Dako), followed by biotinylated peanut agglutinin (Vector Laboratories), ABC-peroxidase Elite (Dako), and chromogenic visualization with diaminobenzidine (Dako). Splenic GC recognized as well-circumscribed clusters of at least 5 PNA^+^ cells were enumerated on one whole splenic section per animal. All sections were visually evaluated in a blinded fashion by a pathologist using a Leica DM6000B microscope equipped with a Leica DFC500 camera and LAS software. Isotype control antibodies were used to confirm antibody specificity for all immunohistochemical stained tissues.

### Renal immune complex immunofluorescence and quantification

Glomerular immune complex deposits were visualized on acetone-fixed 5-micron OCT embedded kidney sections by direct immunofluorescence staining using Alexa Fluor 488-conjugated donkey anti-mu-IgG or goat anti-mu-IgM (Invitrogen); isotype control antibodies were used to confirm antibody specificity for all immunofluorescence stained tissues. Slides were mounted with ProLong Gold Antifade (Invitrogen, Thermo Fisher Scientific), then imaged on a NanoZoomer XR automated slide scanning platform (Hamamatsu) at 200x final magnification. Slide images were analyzed in Matlab (Mathworks), evaluating AF488 channel integrated intensity within manually defined renal cortical regions of interest after correction for background and autofluorescence by scaling and subtracting a non-specific filter channel, cyan fluorescent protein.

### Analysis of serum cytokines and autoantibodies

Serum cytokines were measured using Luminex bead assay (Bio-Rad platform). Total Ig (IgG, IgA, IgM) autoantibodies against mouse nuclear antigens, ds DNA and histones were quantified by mouse anti-nuclear antigens Ig’s ELISA kit, mouse anti-dsDNA ELISA kit and mouse anti-histones Ig’s ELISA kit from Alpha Diagnostic International, according to the manufacture’s protocol.

### Flow cytometry

Single immune cell suspensions were prepared from spleen or peritoneal lavage after red blood cell lysis. Cells were counted on a BD Fortessa Flow cytometer using ‘Fluoresbrite ^TM^ Calibration Grade 6.0 micron YG microspheres (Polysciences). Cells were incubated with anti-CD16/CD32 (Fc block, clone 2.4G2; BD Bioscience) plus 2% normal mouse and normal rat serum and stained with various combinations of the following Abs in FACS buffer (PBS containing 2% FBS and 2 mM EDTA) for 30 min at 4°C. Murine reactive antibodies, including B220-Pacific Blue, CD3-PerCPCy5.5, CD4-Pacific Blue, CD5-PerCPCy5.5, CD8-APC Cy, CD11b APC Cy7, CD21 FITC, CD23 PE, CD38-FITC, CD95 PE Cy7, CD138-PE, IgD APC Cy7, IgM FITC, GL-7 biotin, Streptavidin BV500, Ki67-APC, Ly6G PE Cy7, and Ly6C PerCP Cy5.5 were all purchased from BD Bioscience.

### Western blot

After stimulation of splenic bee cells with 1 mg/ml of R848 for indicated times, cells were washed with cold PBS and lysed with cold lysis buffer (Cell signaling Technology) supplemented with Halt’s protease and phosphatase inhibitors (Thermo Fisher Scientific). Total protein was determined using Pierce BCA protein assay kit. pIRAK4 and pIRAK1 antibodies were generated at Genentech [52], and all other antibodies, including p-IκBα, p-p65 NFκB, p-p38 and p-JNK, actin, and anti-rabbit IgG–HRP linked antibody were purchased from Cell Signaling Technology. Detection was done using SignalFire™ ECL chemiluminescence substrate from Cell Signaling Technology.

### RT PCR of peritoneal exudates

RNA was isolated from frozen peritoneal exudates using the RNeasy mini kit (Qiagen) including the on-column DNase digestion. Total RNA concentration of samples was determined using the NanoDrop 8000 (Thermo Fisher Scientific). cDNA synthesis was performed on 500 ng total-RNA using an iScript cDNA synthesis kit (Bio-Rad). Gene-specific amplification was performed in Real Time PCR System (Applied Biosystem) using Taqman SYBR Green One-Step RT PCR Master Mix (Applied Biosystem) for oasl2, irf7, ifit204, mx-1 and RPL19. The relative abundance (dCt) to RPL19 was calculated and expressed as 2^(avg CT_gene_−avg CT_rpl19_).

### RNA-sequencing

RNA was extracted using Qiagen RNeasy kit per manufacturer’s recommendations (Qiagen). RNA concentration was determined using NanoDrop 8000 (ThermoFisher Scientific), and the integrity of RNA was determined by Fragment Analyzer (Advanced Analytical Technologies). 0.1 μg of total RNA was used as input material for library preparation using TruSeq Stranded Total RNA Library Prep Kit (Illumina). Size of the libraries was confirmed using 4200 TapeStation and High Sensitivity D1K screen tape (Agilent Technologies), and their concentration was determined by qPCR based method using Library quantification kit (KAPA). The libraries were multiplexed and sequenced on Illumina HiSeq4000 (Illumina) to generate 30M of single end 50 base pair reads.

RNA-sequencing analyses were performed using custom scripts written using the R programming language (version 3.5.1) and Bioconductor packages (version 3.8) as detailed below. Raw RNA-sequencing reads were processed using the HTSeqGenie package (version 4.0.1). Reads were aligned to the reference mouse genome (GRCm38) using GSNAP [53], with the following parameters: *-M 2 -n 10 -B 2 -i 1 -N 1 -w 200000 -E 1 –pairmax-rna = 200000 –clip-overlap*. Reads aligning within exon boundaries of gene models (Gencode mouse v. 27) were totaled to yield a per-gene estimate of expression levels. The count data for each experiment were filtered to exclude genes with low expression, retaining genes that had at least 10 reads in at least 4 samples. We used the voom package [54] to normalize expression data, and the limma package [55, 56] to calculate differential expression across groups. For transcriptome-wide analyses, we used the method of Benjamini and Hochberg [57] to adjust for multiple testing. Genes were considered differentially expressed if they were at least 2-fold different between groups, at a false discovery rate (FDR) of 5%. For gene set enrichment analyses, we used gene sets as previously defined [58], using the roast method [59] from the limma package to find gene sets that were enriched in specific comparisons.

### Statistical analysis and graphical representation

Statistical analysis and graphing were done using GraphPad Prism (7.0) software. Comparison for each pair was performed using two-tailed Student’s *t*-test with Welch’s correction; multiple comparisons with a single control were performed using one-way ANOVA with Dunnett’s correction or Kruskal-Wallis test with Dunn’s correction. Survival plots were analyzed by log-rank Mantel-Cox test. *p*-values < 0.05 were considered significant. For histopathology and Immunofluorescence, nonparametric (glomerulonephritis, tubule-interstitial nephritis, and periarteritis) and parametric (# germinal centers) statistical group comparisons were performed in Prism 6 backwards and forwards by the nonparametric Kruskal-Wallis test with Dunn’s multiple comparisons test, and by the parametric ANOVA test with Holm-Sidak correction for multiple comparisons. Statistically significant differences are denoted as * p<0.05, **p<0.005 and ***p<0.0005, non-significant data is left without an Asterix.

## Supporting information

S1 Fig SLC15a4 regulates expression of interferon signature genes, expression of inflammatory cytokines and is dispensable for TLR4 induced cytokine production in B cells. B cells were stimulated with 1μg/ml R848, and gene expression was analyzed 4 or 24 hours later. (A-B) Calculated fold- change slc15a4^-/-^ (on C57BL/6 background) (x-axis) and wildtype B cells (C57BL/6) (y-axis) post 4hr (A) and 24hr (B) stimulations. Red, green and blue dots represent genes that are significant only in naive versus diseased condition in wildtype (red), only in knockout versus diseased wildtype (green) or both (blue). (C) Heatmap visualization of interferon regulated genes (log fold change >1.5, p<0.05). (D-G) representative box plots of individual interferon signature genes. (H) Heatmap visualization of NFκB dependent genes (log fold change >1.5, p<0.05). (I-L) representative box plots of individual NFκB dependent genes. (M-N) Heatmap visualization of inflammatory cytokine genes (M) and plasma cell differentiation genes (N) (log fold change >1.5, p<0.05). (O-Q) B cells were stimulated with LPS and analyzed for cytokine expression by Luminex analysis of supernatants at 72 hours. N = 3 and cytokine data is expressed as mean ± SD.
(TIF)

S2 Fig SLC15a4 is dispensable for TLR4 induced cytokine production in myeloid cells. Cytokine production upon LPS stimulation for 24 hours was analyzed by Luminex in myeloid dendritic cells (A-E) and macrophages(F-J) from slc15a4^-/-^ (on C57BL/6 background) and wildtype (C57BL/6) mice. Data is expressed as mean ± SD, N = 3.
(TIF)

S3 Fig Disruption of the slc15a4 gene and validation of knockout in NZB and NZW strains. CRISPR mediated targeting resulted in an 8 bp deletion in NZW mice, and a 12609 bp deletion in NZB mice (A, B). Western blot analysis of spleens from *slc15a4*^*+/+*^, *slc15a4*^*+/-*^ (NZB with the 12609 bp deletion), and *slc15a4*^*-/-*^ mice demonstrates absence of SLC15a4 protein (C). Functional absence was demonstrated by stimulation of splenic B cells from *slc15a4*^*+/+*^, *slc15a4*^*+/-*^ and *slc15a4*^*-/-*^ mice with R848 (D), ODN2006 (E), and LPS (F), resulting in diminished IL-6, and by stimulation of plasmacytoid dendritic cells with ODN2216, resulting in complete absence of IFNα production (G). Cytokine production was assessed by ELISA kits for IL-6 and IFNα (R&D Biosciences) in triplicates and expressed as mean ± SD. Comparisons were made between wildtype group and knockouts or heterozygous groups *p < 0.05, **p<0.005, ***p<0.0005, ****p<0.0001, ns, not significant.
(TIF)

S4 Fig SLC15a4 deficient NZB/W F1 mice have reduced serum IgG, IgM and IgA. Serum concentrations of Ig isotypes at 13 and 32 weeks of age was determined by Luminex. Data is expressed as mean ± SD, N = 4–11 per group. Comparisons were made between wildtype group and knockouts or heterozygous groups *p < 0.05, **p<0.005.
(TIF)

S5 Fig Design of a combined survival/biomarker study in IFNα accelerated NZB/W F1 mice. Mice were randomly assigned to either a survival cohort or a biomarker cohort. No NZB/W F1 *slc15a4*^*+/-*^ mice were assigned to the survival cohort due to limited availability. All mice received 1.2x10^8^ pfu rAd5- IFNα i.v. on day 0, and all mice were bled on days 21 and 40 for serum analysis. The biomarker cohort was terminated on day 47, and the survival cohort was terminated on day 154. Endpoints are shown in bold blue type.
(TIF)

S1 Raw images (PDF)
