# Supplementary figures and images for: The peptide symporter SLC15a4 is essential for the development of systemic lupus erythematosus in murine models
Source: PLoS One. 2021 Jan 14;16(1):e0244439. doi: 10.1371/journal.pone.0244439 (PMC7808665; doi:10.1371/journal.pone.0244439)

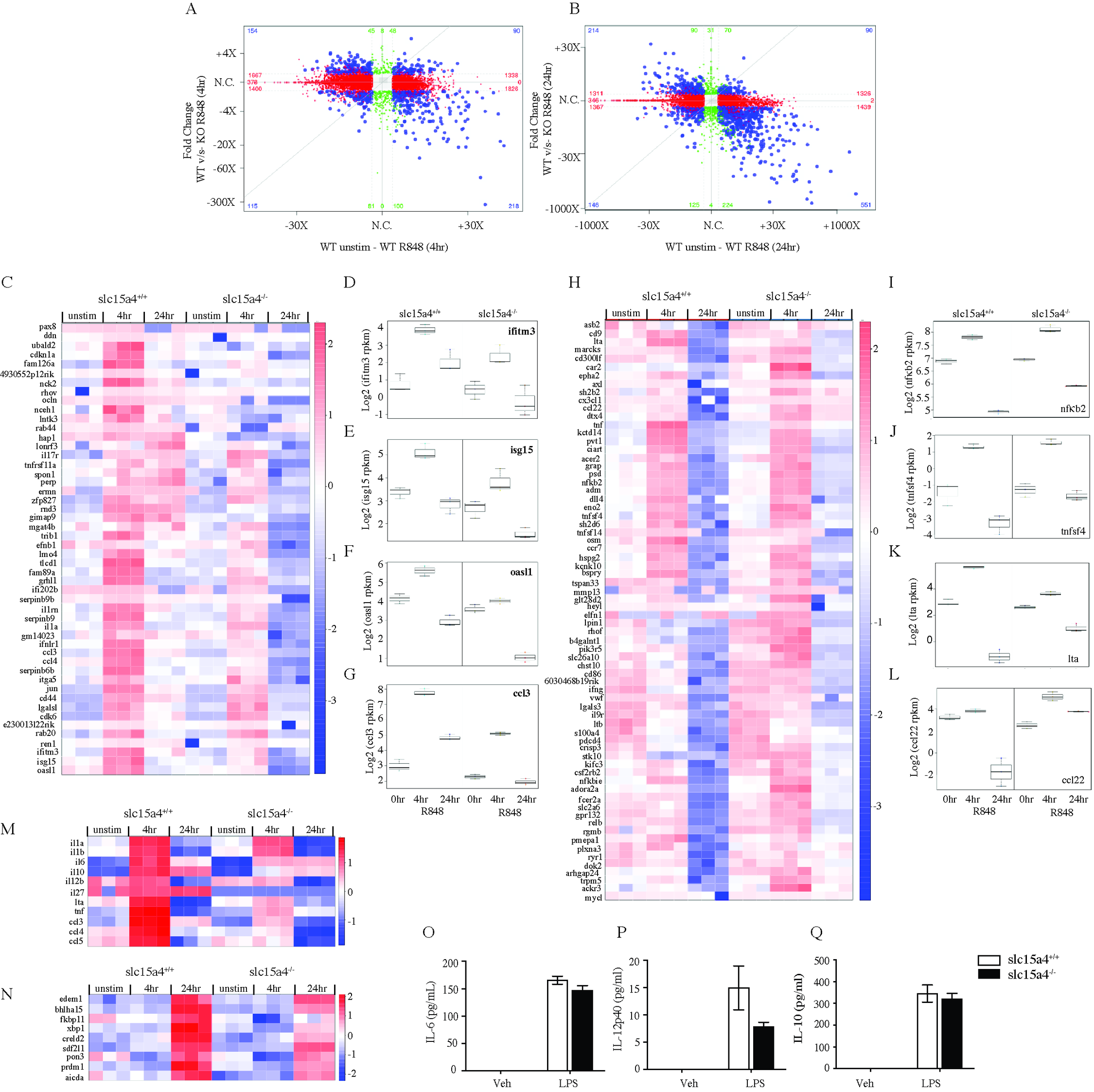

Supplement: S1 Fig — B cells were stimulated with 1μg/ml R848, and gene expression was analyzed 4 or 24 hours later. (A-B) Calculated fold- change slc15a4-/- (on C57BL/6 background) (x-axis) and wildtype B cells (C57BL/6) (y-axis) post 4hr (A) and 24hr (B) stimulations. Red, green and blue dots represent genes that are significant only in naive versus diseased condition in wildtype (red), only in knockout versus diseased wildtype (green) or both (blue). (C) Heatmap visualization of interferon regulated genes (log fold change >1.5, p<0.05). (D-G) representative box plots of individual interferon signature genes. (H) Heatmap visualization of NFκB dependent genes (log fold change >1.5, p<0.05). (I-L) representative box plots of individual NFκB dependent genes. (M-N) Heatmap visualization of inflammatory cytokine genes (M) and plasma cell differentiation genes (N) (log fold change >1.5, p<0.05). (O-Q) B cells were stimulated with LPS and analyzed for cytokine expression by Luminex analysis of supernatants at 72 hours. N = 3 and cytokine data is expressed as mean ± SD. (TIF) [file pone.0244439.s001.tif]

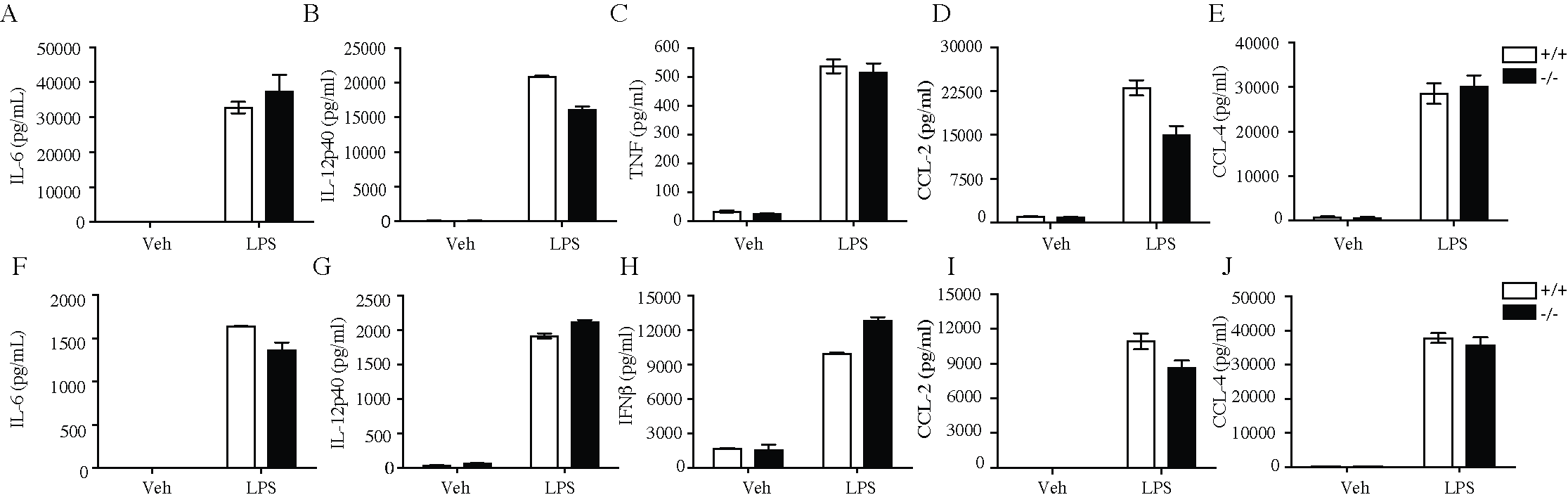

Supplement: S2 Fig — Cytokine production upon LPS stimulation for 24 hours was analyzed by Luminex in myeloid dendritic cells (A-E) and macrophages(F-J) from slc15a4-/- (on C57BL/6 background) and wildtype (C57BL/6) mice. Data is expressed as mean ± SD, N = 3. (TIF) [file pone.0244439.s002.tif]

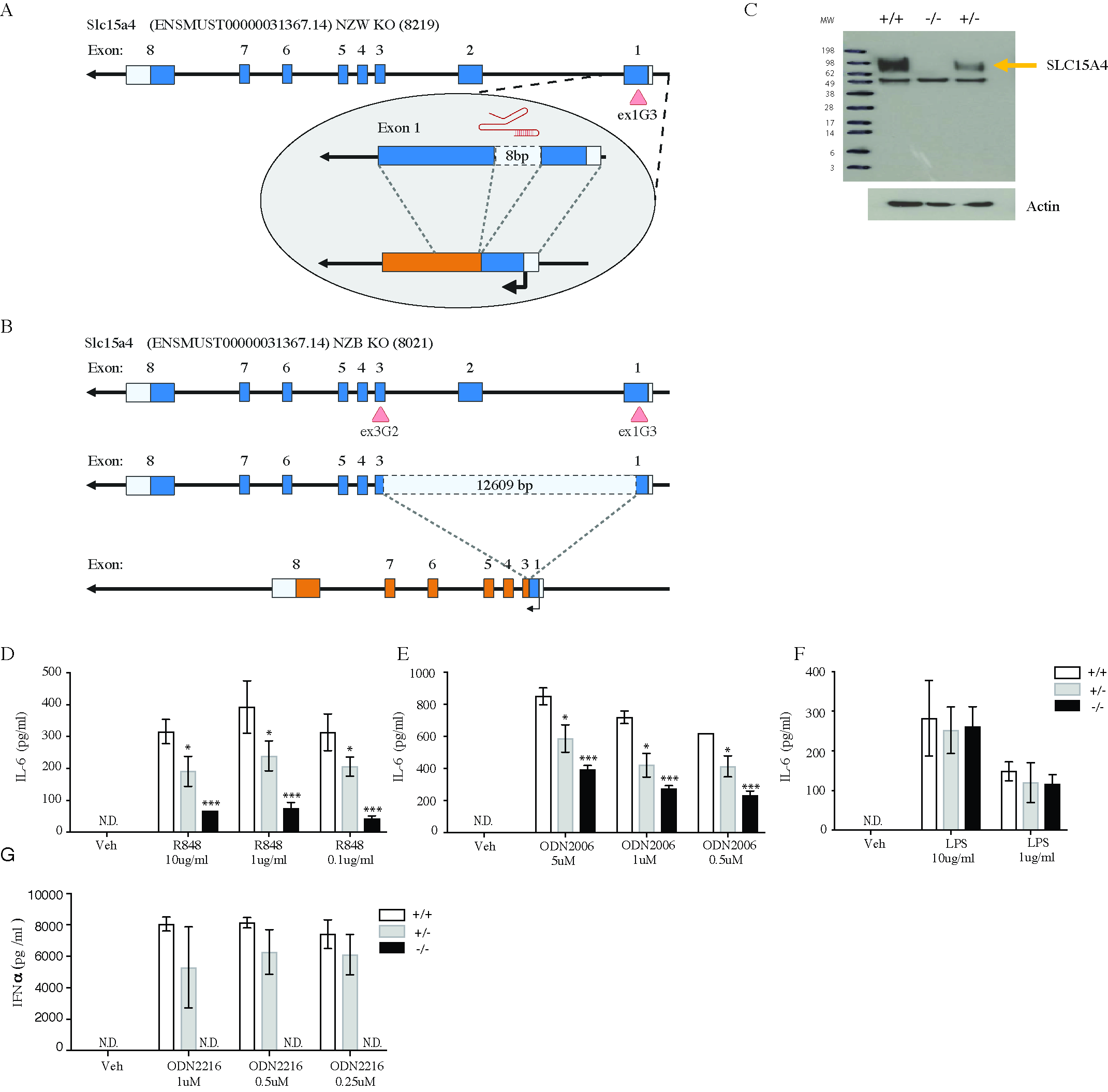

Supplement: S3 Fig — CRISPR mediated targeting resulted in an 8 bp deletion in NZW mice, and a 12609 bp deletion in NZB mice (A, B). Western blot analysis of spleens from slc15a4+/+, slc15a4+/- (NZB with the 12609 bp deletion), and slc15a4-/- mice demonstrates absence of SLC15a4 protein (C). Functional absence was demonstrated by stimulation of splenic B cells from slc15a4+/+, slc15a4+/- and slc15a4-/- mice with R848 (D), ODN2006 (E), and LPS (F), resulting in diminished IL-6, and by stimulation of plasmacytoid dendritic cells with ODN2216, resulting in complete absence of IFNα production (G). Cytokine production was assessed by ELISA kits for IL-6 and IFNα (R&D Biosciences) in triplicates and expressed as mean ± SD. Comparisons were made between wildtype group and knockouts or heterozygous groups *p < 0.05, **p<0.005, ***p<0.0005, ****p<0.0001, ns, not significant. (TIF) [file pone.0244439.s003.tif]

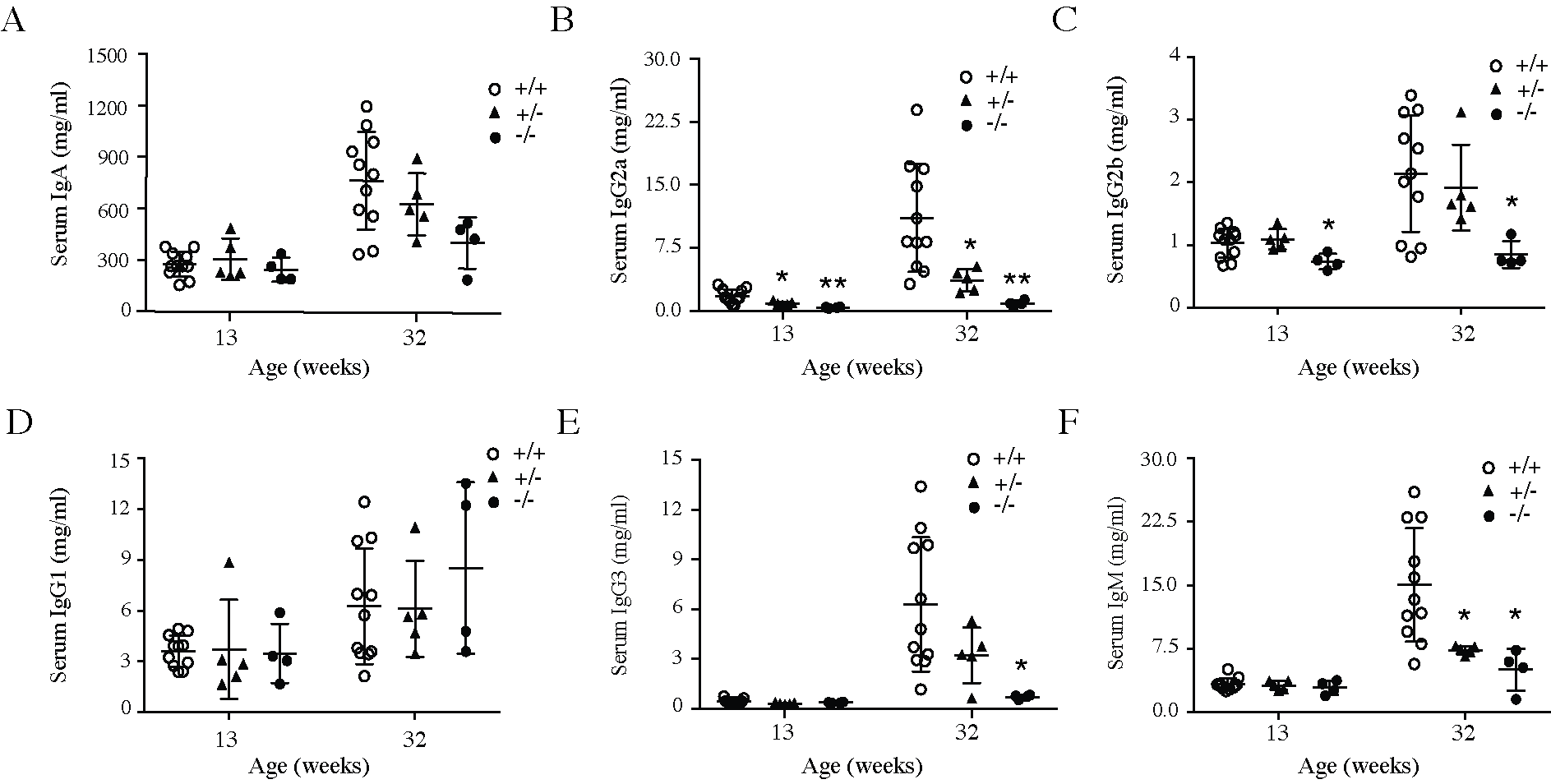

Supplement: S4 Fig — Serum concentrations of Ig isotypes at 13 and 32 weeks of age was determined by Luminex. Data is expressed as mean ± SD, N = 4–11 per group. Comparisons were made between wildtype group and knockouts or heterozygous groups *p < 0.05, **p<0.005. (TIF) [file pone.0244439.s004.tif]

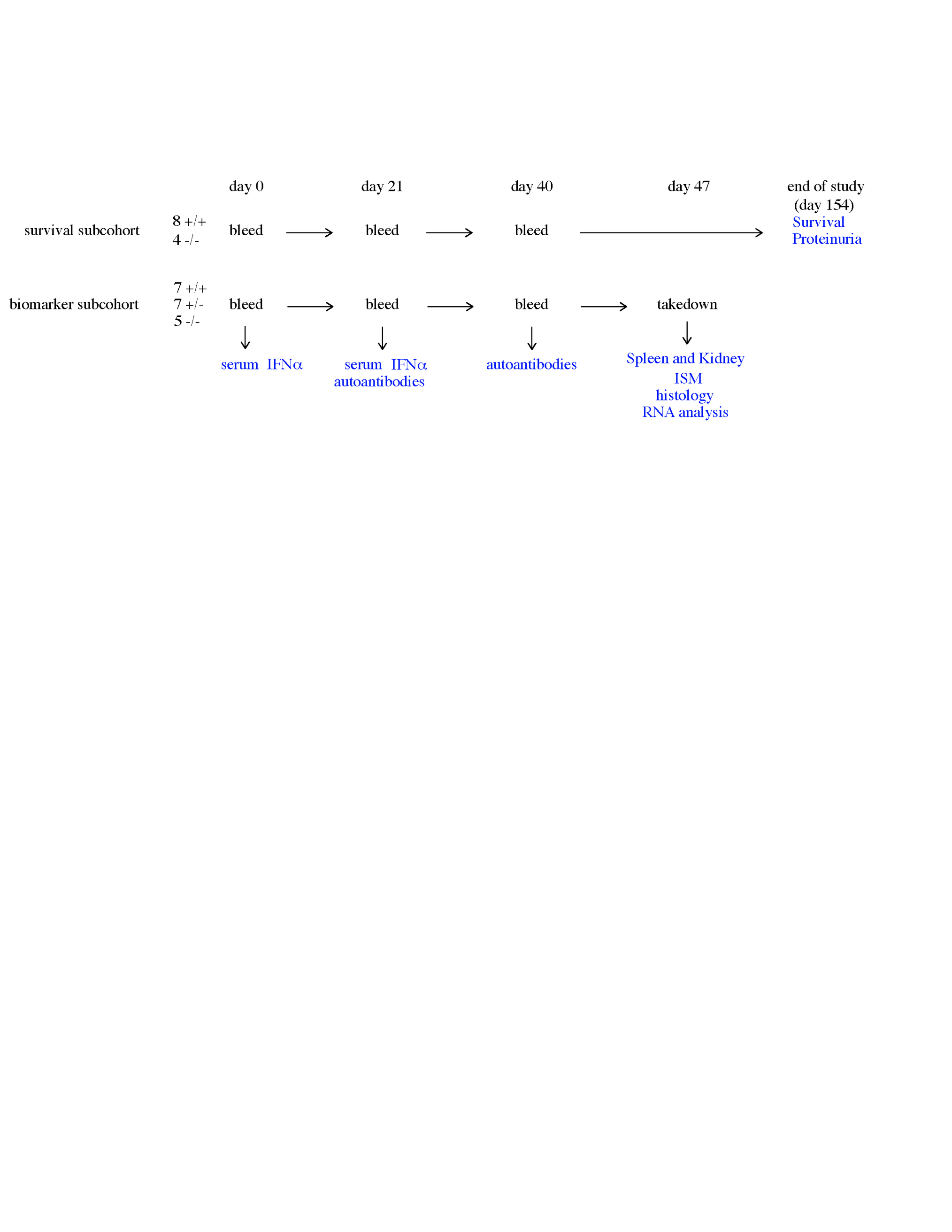

Supplement: S5 Fig — Mice were randomly assigned to either a survival cohort or a biomarker cohort. No NZB/W F1 slc15a4+/- mice were assigned to the survival cohort due to limited availability. All mice received 1.2x10^8^ pfu rAd5- IFNα i.v. on day 0, and all mice were bled on days 21 and 40 for serum analysis. The biomarker cohort was terminated on day 47, and the survival cohort was terminated on day 154. Endpoints are shown in bold blue type. (TIF) [file pone.0244439.s005.tif]
