# Supplementary material for: The peptide symporter SLC15a4 is essential for the development of systemic lupus erythematosus in murine models
Source: PLoS One. 2021 Jan 14;16(1):e0244439. doi: 10.1371/journal.pone.0244439 (PMC7808665; doi:10.1371/journal.pone.0244439)
Supplement: S1 Raw images — (PDF) [file pone.0244439.s006.pdf]

$slc15a4^{+/+}$ $slc15a4^{-/-}$

p-IRAK4 M 0’ 15’ 30’ 60’ 120’ 0’ 15’ 30’ 60’ 120’

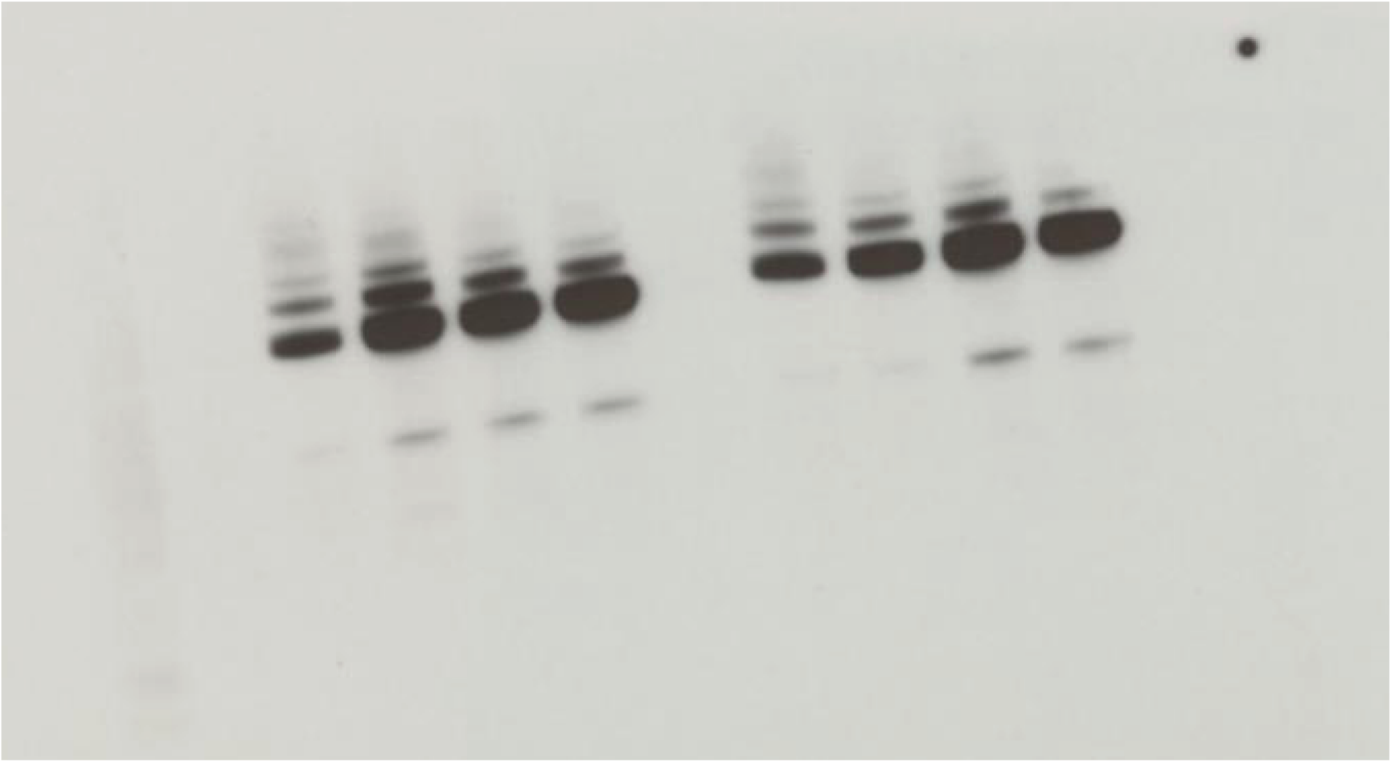

$slc15a4^{+/+}$ $slc15a4^{-/-}$

p-IRAK1 M 0’ 15’ 30’ 60’ 120’ 0’ 15’ 30’ 60’ 120’

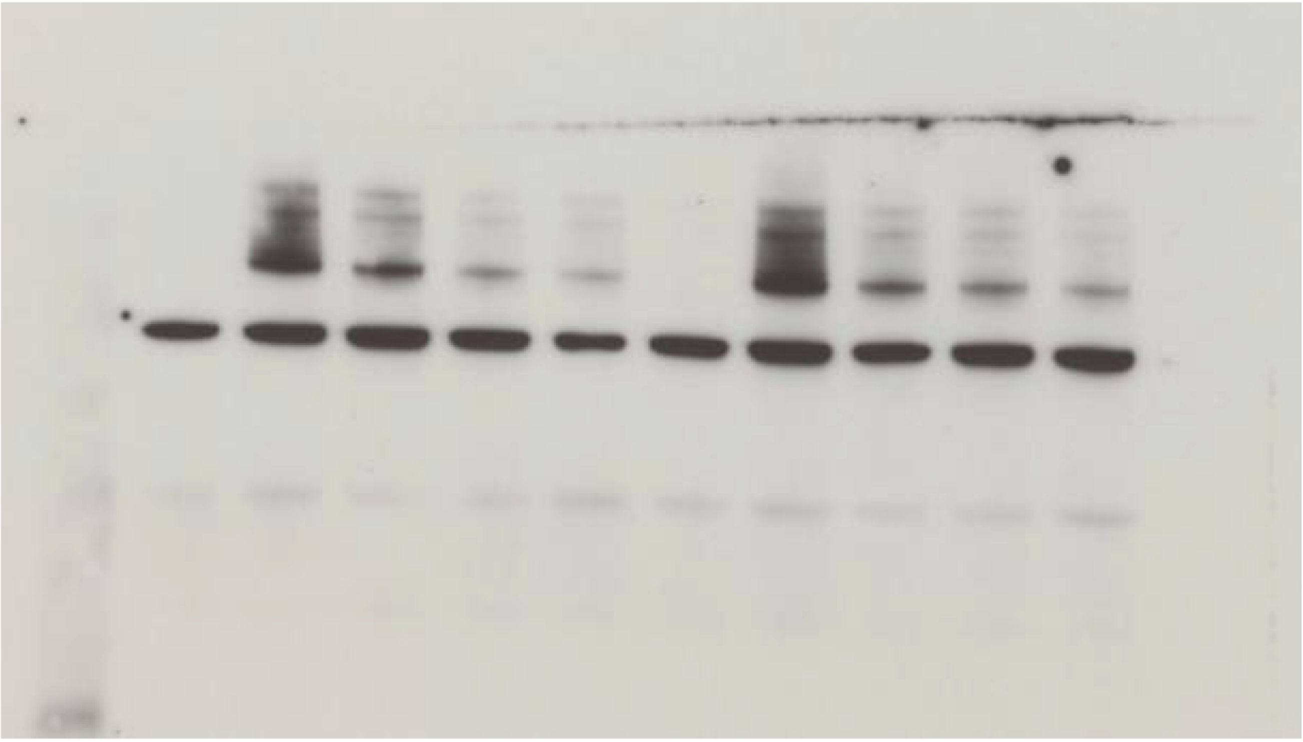

Figure 2F

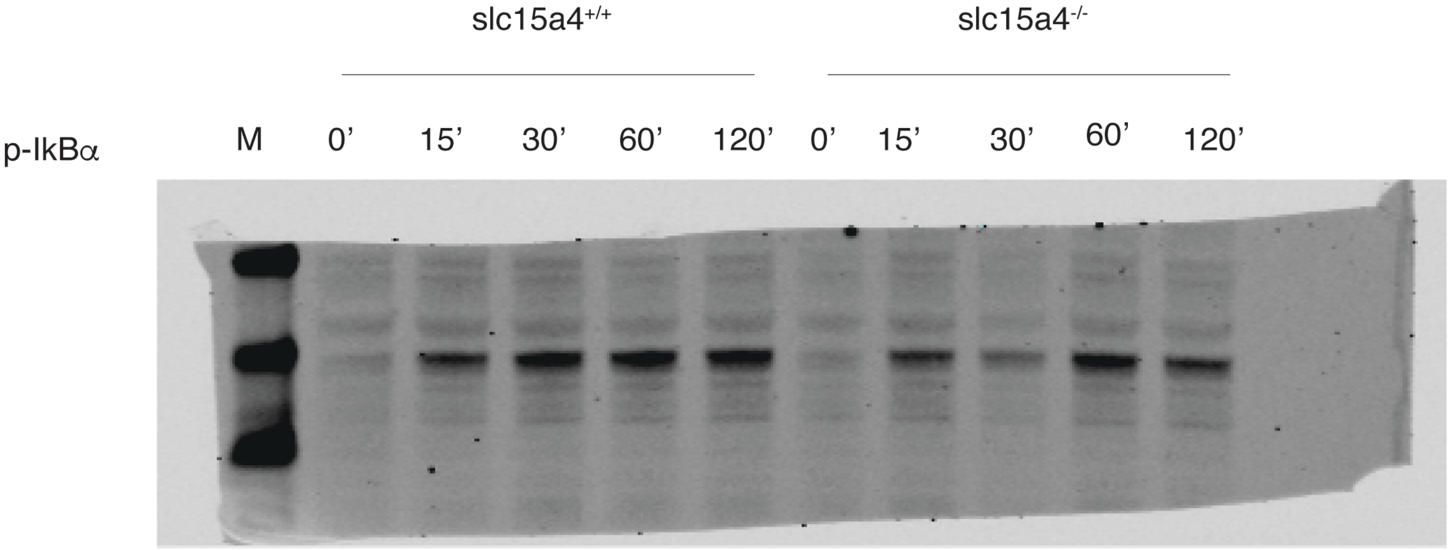
slc15a4+/+
slc15a4-/-
p-IkBα
M
0’
15’
30’
60’
120’
0’
15’
30’
60’
120’

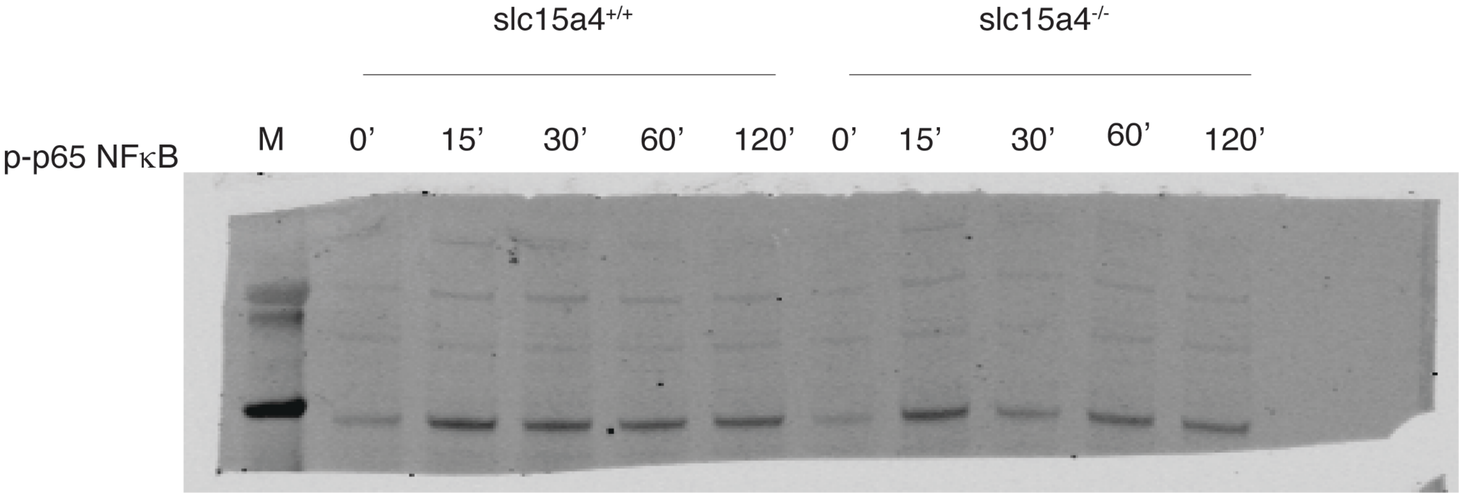
slc15a4+/+
slc15a4-/-
p-p65 NFκB
M
0’
15’
30’
60’
120’
0’
15’
30’
60’
120’

Figure 2F

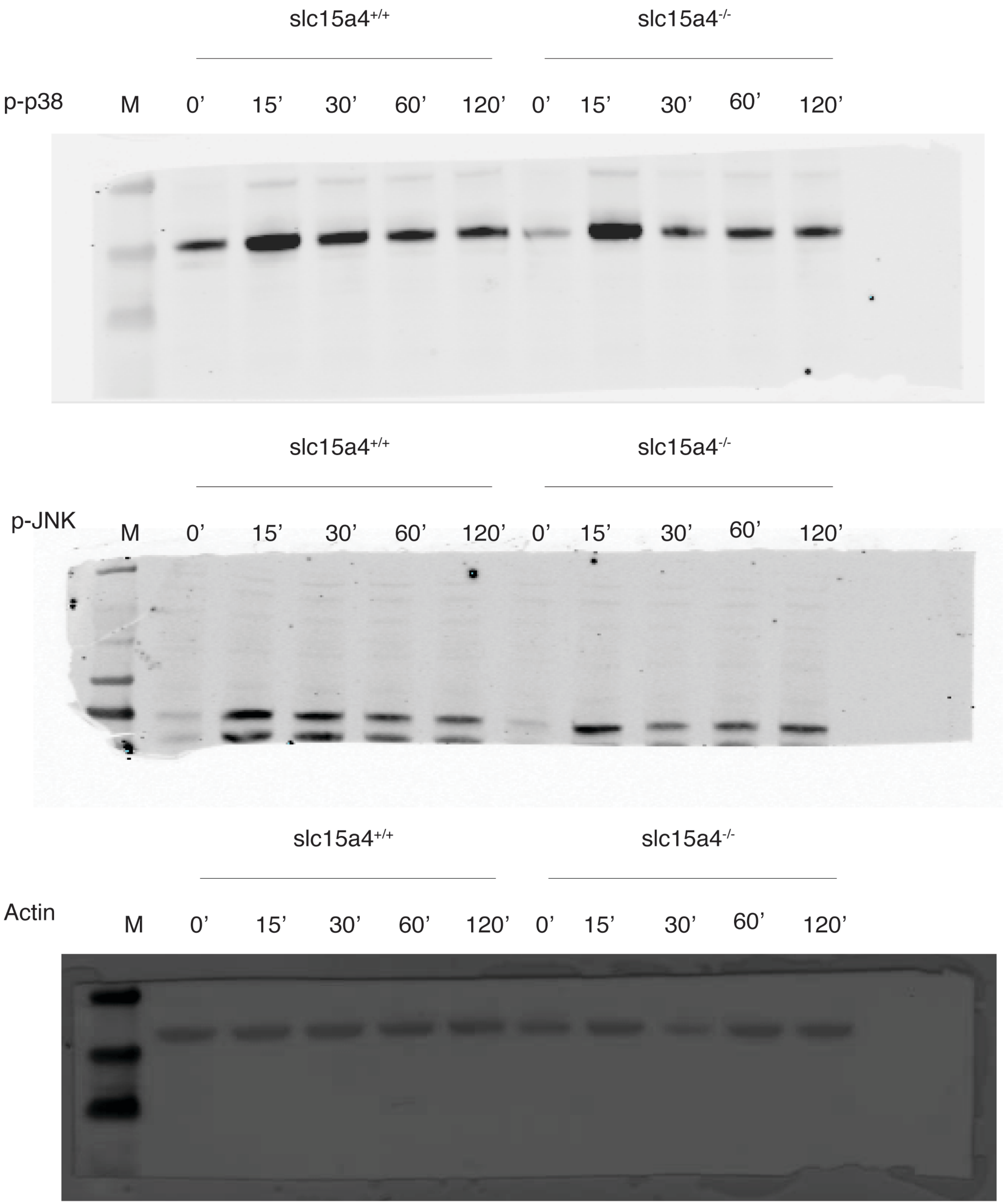
slc15a4+/+
slc15a4-/-
p-p38
M
0’
15’
30’
60’
120’
0’
15’
30’
60’
120’
slc15a4+/+
slc15a4-/-
p-JNK
M
0’
15’
30’
60’
120’
0’
15’
30’
60’
120’
slc15a4+/+
slc15a4-/-
Actin
M
0’
15’
30’
60’
120’
0’
15’
30’
60’
120’

Figure 2F

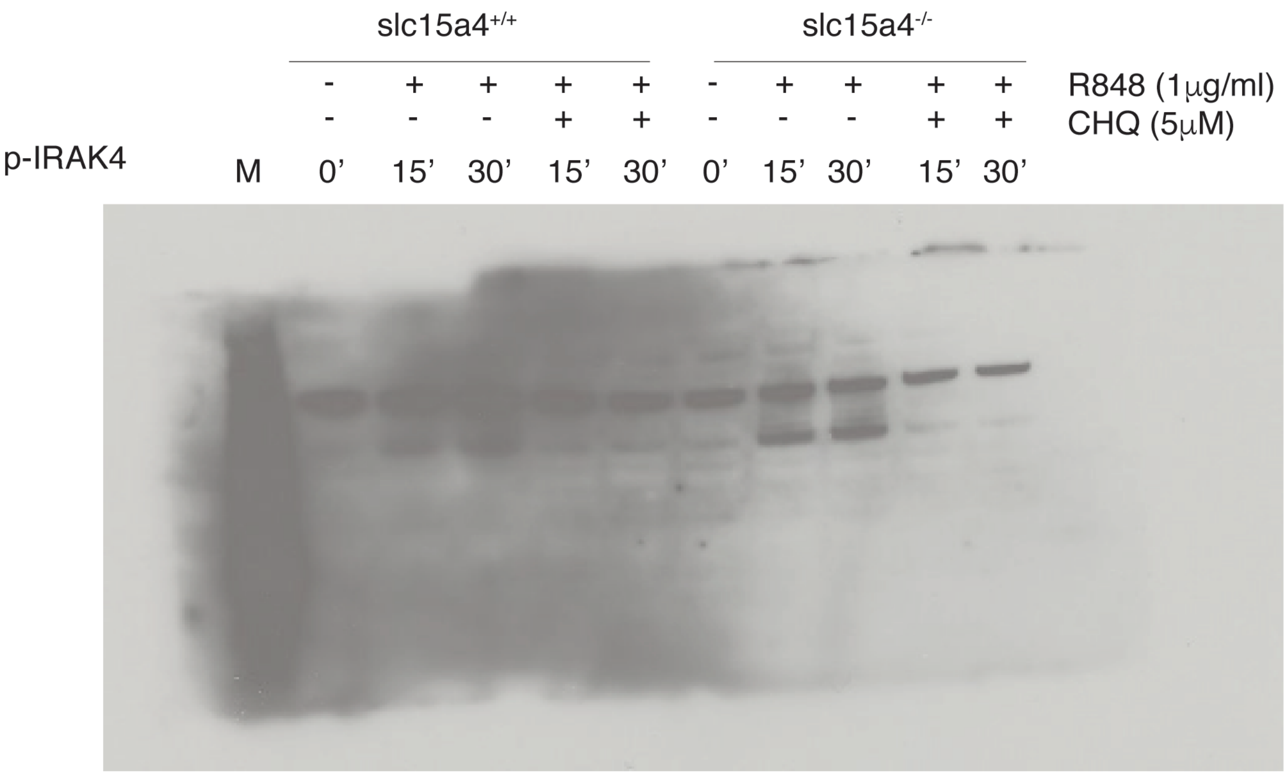
slc15a4+/+
slc15a4-/-
- + + + + - + + + +
R848 (1μg/ml)
- - - + + - - - + +
CHQ (5μM)
p-IRAK4
M 0' 15' 30' 15' 30' 0' 15' 30' 15' 30'

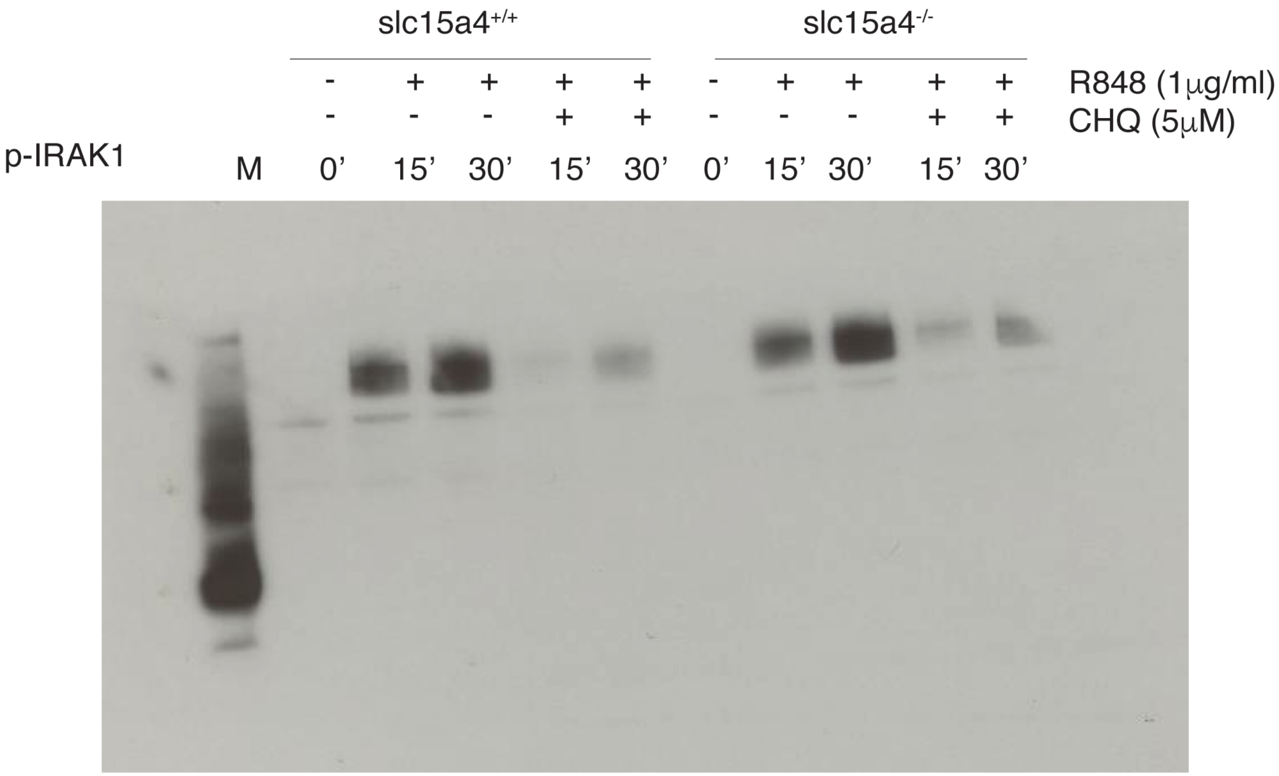
slc15a4+/+
slc15a4-/-
- + + + + - + + + +
R848 (1μg/ml)
- - - + + - - - + +
CHQ (5μM)
p-IRAK1
M 0' 15' 30' 15' 30' 0' 15' 30' 15' 30'

Figure 2G

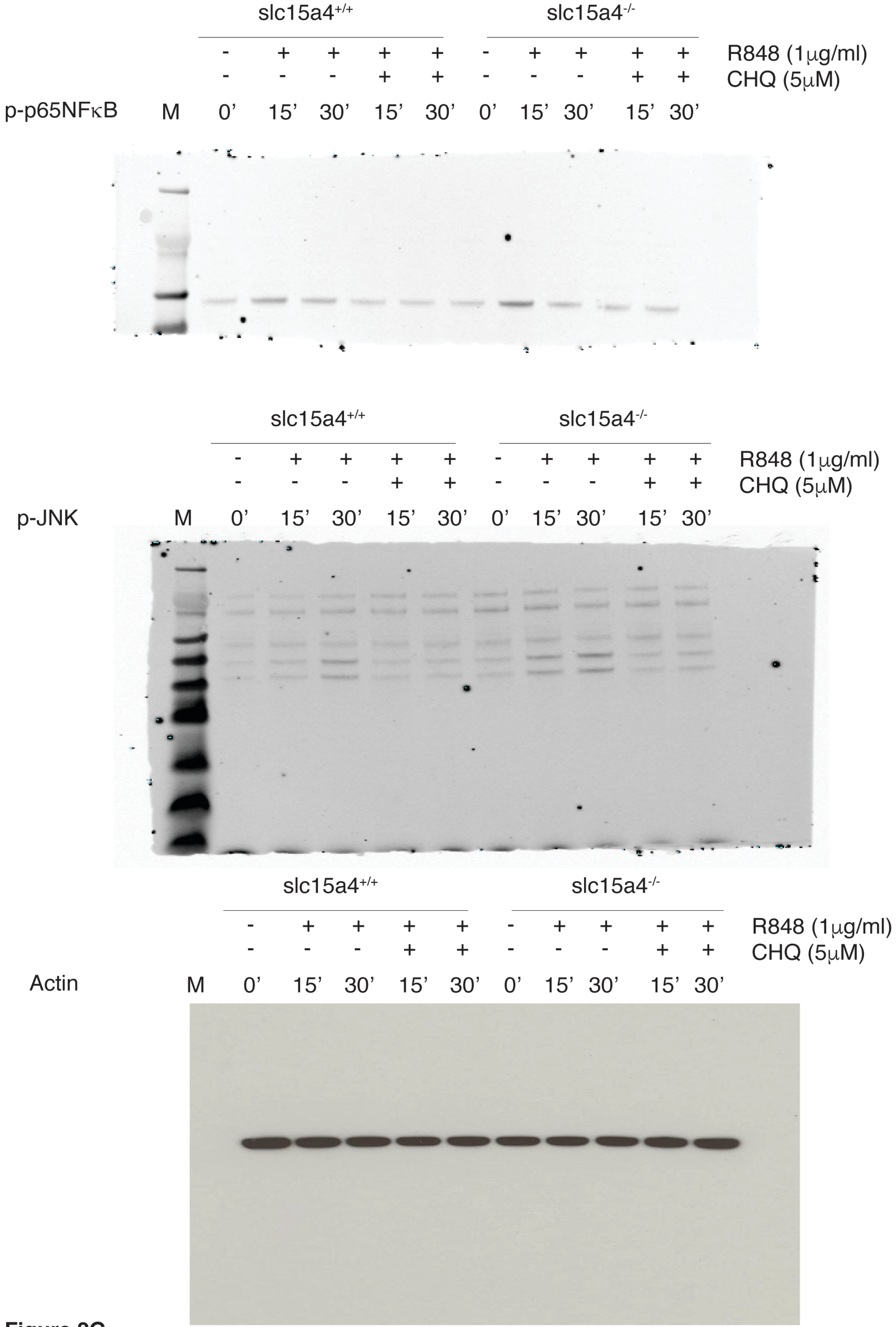
slc15a4+/+
slc15a4-/-
R848 (1μg/ml)
CHQ (5μM)
p-p65NFκB
M
0' 15' 30' 15' 30' 0' 15' 30' 15' 30'
slc15a4+/+
slc15a4-/-
R848 (1μg/ml)
CHQ (5μM)
p-JNK
M
0' 15' 30' 15' 30' 0' 15' 30' 15' 30'
slc15a4+/+
slc15a4-/-
R848 (1μg/ml)
CHQ (5μM)
Actin
M
0' 15' 30' 15' 30' 0' 15' 30' 15' 30'

Figure 2G
